# Bibliometric and literature review of research on nature-based solutions and climate change: Implications for policy and practice

**DOI:** 10.1007/s13280-025-02273-y

**Published:** 2025-10-29

**Authors:** Carmen Echebarria, Izaskun Gomez de Salazar

**Affiliations:** 1 Research Group on Governance and Marketing for Sustainability (2022–2025), Consolidated Group of the Basque Research System (IT1731-22), Department of Applied Economics, Faculty of Economics and Business, University of the Basque Country (UPV/EHU), Avenida Lehendakari Agirre 83, 48015 Bilbao, Spain; 2 Research Group on Governance and Marketing for Sustainability (2022–2025), Consolidated Group of the Basque Research System (IT1731-22), University of the Basque Country (UPV/EHU), C/Tomás y Valiente s/n, 01006 Vitoria-Gasteiz, Spain

**Keywords:** Bibliometric analyses, Climate change, Literature review, Nature-based solutions

## Abstract

Nature-based solutions to adapt to climate change have attracted increasing interest in recent years and have developed in different directions. This study aims to provide an updated overview of this growing field, its main trends, and directions for future research. To this end, we conducted several analyses. First, we selected 258 papers from the Web of Science database, published between 2009 and 2023, and presented their profiles in terms of time, journals, geography, and research areas. Second, we performed a bibliometric co-word analysis, which identified four thematic clusters: (1) urban planning, (2) disaster risk reduction, (3) forest, and (4) biodiversity, providing a holistic view of the field. Third, we supplemented the bibliometric analyses with a literature review, to help interpret the themes in each thematic cluster and identify potential avenues for future research. We hope that this review will provide valuable information as a guide for both academics and practitioners.

**Supplementary Information:**

The online version contains supplementary material available at 10.1007/s13280-025-02273-y.

## Introduction

Today’s societies face major challenges such as climate change, rapid urbanization, natural disaster risk, deforestation, food security, and water resource supply (Cohen-Shacham et al. 2019). One approach to respond to these challenges is to manage social-ecological systems holistically to sustain, and even enhance, the provision of ecosystem services to human populations (Eggermont et al. 2015). Based on prior literature (e.g., Raymond et al. 2017; Cohen-Shacham et al. 2019; Frantzeskaki, 2019), we define nature-based solutions as nature-inspired and nature-supported ecosystem interventions aimed at addressing socio-environmental challenges, particularly climate change. Also based on previous literature (e.g., Blackwood and Renaud, 2022; Duffaut et al. 2022; Seddon, 2022), by climate change we mean the increase in temperature, changes in precipitation patterns, intensification of extreme weather events, and loss of biodiversity. According to Nassary (2025), the key principles guiding nature-based solutions include promoting ecosystem health (Cohen-Shacham et al. 2019), respecting local communities and indigenous knowledge, ensuring long-term viability, and integrating other sustainable development goals into the solutions themselves with other sustainable development goals (Kabisch et al. 2022).

Nature-based solutions thus represent an ambitious and appealing approach to addressing contemporary socio-environmental challenges by integrating ecological processes with societal needs (Cohen-Shacham et al. 2016; Seddon et al. 2021; Nassary 2025). However, despite their relevance, the practical application of, and research into, nature-based solutions as a way of tackling socio-environmental challenges, particularly climate change, remain scarce and fragmented (Chausson et al. 2020). It should be noted that this is a relatively new field of research, which has started to gain importance in the last two decades. This situation has led to the development of studies on diverse and scattered themes, highlighting the need to synthesize and classify the existing literature. In this regard, several previous studies have reviewed the literature on nature-based solutions and climate change (see Table 1), with the aim of understanding the development of this field of research.

**Table 1:** Summary of extant bibliometric reviews on nature-based solutions and climate change

Authors (year)	Source	Key contributions	Data base	Methodology	Period
Liu et al. (2021)	*Building and Environment*	This article describes the evolution of scientific research on implementing green roofs between 1981 and 2019 and its impact on regulating water, temperature and air quality, using the Bibliometrix and CiteSpace R packages	Scopus (1623 papers)	Systematic review Bibliometric	1981–2019
Cousins (2021)	*Ecological Economics*	This study maps out the structure of academic research on nature-based solutions and finds that issues of social and environmental justice remain on the periphery. It proposes a shift toward just nature-based solutions. Bibexcel and Gephi were used	Web of Science (188 papers)	Content analysis Systematic review	Up to 2019
Matsler et al. (2021); Bunclark and De la Vega Hernández (2022); Zhong et al. (2023)	*Landscape and Urban Planning* *Water Resources Management* *Ecological Indicators*	This article conducts a systematic review to understand how green infrastructure is conceptualized in many fields such as urban planning, urban forestry, ecology, engineering, landscape architecture and law, using Bibliometrix R package and computer-assisted qualitative data analysis (CAQDAS) techniques with ATLAS.ti software This article provides an analysis and systematization of the literature on nature-based solutions for managing water resources. It helps to identify research gaps and potential areas for future research. The visualization programs applied were VOSviewer and Bibliometrix, and the statistical tools used were R and Python This paper explores the hotspots and trends in blue carbon research, using the Bibliometrix package in R studio and VOSviewer software, to guide future research on mitigating climate change and achieve carbon removal and neutrality goals	Scopus (75 papers), Web of Science (1422 papers), and analysis of document type and field for top 30 Google hits for green infrastructure Web of Science (8199 papers) Web of Science Core Collection (2613 papers)	Multi-method approach (content analysis, systematic review, and exploration grey literature review) Systematic Review Bibliometric Systematic Review Bibliometric	1990–2018 1990–2018 2003 to 2021

Despite their relevance, these previous reviews focused on specific aspects of the topic and have left gaps in our overall understanding of the knowledge structure of research on nature-based solutions and climate change. For example, Liu et al. (2021) focused on the impact of green roofs on regulating water, temperature, and air quality, while the review by Zhong et al. (2023) explored hotspots and trends in blue carbon research. In their study, Matsler et al. (2021) conducted a systematic review to understand how green infrastructure is conceptualized in different disciplines using a multi-method approach (content analysis, bibliometric review, and exploratory grey literature review). Bunclark and De la Vega Hernández (2022) analyzed and systematized scientific literature related to nature-based solutions for managing water resources, while Cousins (2021) highlighted the lack of social and environmental justice issues in nature-based solutions, calling for a shift toward just nature-based solutions. Although these studies have made significant contributions to this body of research, their fragmentation is a limitation to a complete understanding of the research area. This fragmentation of the literature is common in relatively new research fields that have yet to reach maturity. Moreover, the time factor must be considered. The number of publications and topics on nature-based solutions and climate change has increased considerably, especially since 2020, so it is necessary to keep track of how the field is developing.

To address these limitations and build on the insights provided, this study aims to give a structured overview of the field of research, explore the main research topics, and guide future research. To this end, three related research questions have been formulated: (1) What is the trend regarding the number of publications on nature-based solutions and climate change? (2) What are the main research topics underpinning the development and growth of the field? (3) What are the emerging research trends?

To this end, we carried out a two-tier methodological approach consisting of a bibliometric analysis and a literature review of papers on nature-based solutions and climate change, identified in the Clarivate Analytics Web of Science (WoS) database for the period 2009–2023. On the one hand, bibliometric tools allow us to carry out a descriptive analysis using bibliometric performance indicators to identify key publication patterns and provide an up-to-date overview of the research field. On the other, through a co-word analysis, they also enable us to produce a strategic diagram to graphically illustrate current research themes, emerging topics, and potential trends for future research. After mapping the knowledge domain of the field using a co-word analysis, we complemented our study with a literature review, which involved analyzing and interpreting the bibliometric map. The combination of these two methods is regarded as an appropriate choice for analyzing emerging and fragmented research fields (Tripathy et al. 2024).

On this basis, the objectives of this study are: (1) to show the growth and evolution of the topic over the study period; (2) to shed light on the field’s current areas of interest through a co-word analysis, to allow us to identify clusters representing the latest research themes in the field of nature-based solutions and climate change; and (3) to identify research topics that warrant further attention, thereby providing insights into potential avenues for future research through an in-depth review of the papers included within each thematic cluster, as revealed by the bibliometric approach.

This study contributes to the literature in several ways. First, it synthesizes and organizes existing knowledge about nature-based solutions and the climate change research field through a bibliometric review of 258 papers published from 2009 to 2023. In doing so, it provides an updated overview of this growing research field, outlining its profile in terms of time, journals, geography, and research areas. Second, it identifies the conceptual structure of the research field through a co-word analysis. This analysis reveals that the research field on nature-based solutions and climate change is organized into four thematic clusters: (1) urban planning, (2) disaster risk reduction, (3) forests, and (4) biodiversity. Third, based on the results obtained from the co-word analysis, this study provides avenues for developing the research field through an in-depth review of the papers included in each thematic cluster. This approach allows us to identify the main findings and knowledge gaps and propose a future research agenda for the field. Therefore, this study contributes to a better understanding of the conceptual and intellectual structure of research on nature-based solutions and climate change, based on a global overview of the relevant literature.

The remainder of this paper is structured as follows. Section "Research methodology" describes the research methodology used in this study. Section "Results" This stage is where the results from the bibliometric study are presented provides an overall profile of the papers in terms of time, journals, geography, and research areas. Section "Co-word analysis" identifies four thematic clusters based on a co-word analysis, while Sect. "Literature review" provides an in-depth analysis of the papers within each cluster. The last section concludes with a brief overview of our findings, key considerations, limitations, and suggestions for future research.

## Research methodology

We followed a methodology with three sequential steps to conduct our review on existing publications in the field of nature-based solutions and climate change (e.g., Patton 2002): (1) defining the inclusion criteria for studies; (2) setting the strategy for locating and selecting the studies for potential inclusion; and (3) choosing the methods for organizing and analyzing the selected studies. Consequently, we adopted a two-tiered methodological approach involving a bibliometric analysis and a literature review. In the first stage, we conducted a bibliometric analysis using quantitative techniques to examine publication patterns in the field of study. Bibliometric tools allowed us to systematically organize all the extant scientific activity and provide a comprehensive knowledge map of the field (Callon et al. 1986; Cobo et al. 2011a). Following the recommendations of Cobo et al. (2011a), we applied clustering algorithms to facilitate the selection, organization, and visualization of information, allowing us to represent the field in a structured manner. After mapping the knowledge domain using co-word analysis, we carried out a systematic literature review following a clearly defined protocol to reduce bias and ensure transparency (Tranfield et al. 2003; Jalonen 2012; Sutton et al. 2019). In this phase, we rigorously analyzed the selected studies and integrated their findings. This process allowed us to develop a critical, evidence-based synthesis of the state of knowledge in the field, providing a deeper perspective that complements the overview derived from the bibliometric analysis. Therefore, systematic literature reviews not only provide reliable insights for advancing academic research but also serve as a valuable foundation for evidence-based practice and policymaking. The complementarity of these two methods highlights their suitability for analyzing interdisciplinary and emerging research fields, such as nature-based solutions and climate change, and is in line with the types of reviews proposed by Sutton et al. (2019), who recognize the validity of hybrid approaches for studying such fields.

### Inclusion criteria

Three inclusion criteria were used as a guide for selecting and assessing the studies for potential inclusion. A study had to meet three conditions to be included in the systematic review:

1. It must be published as an article or review in a peer-review scientific journal.
2. It must be published during the period 2009-May 2023.
3. It must be written in English.

### Search strategy and selection procedure

**Fig. 1:**
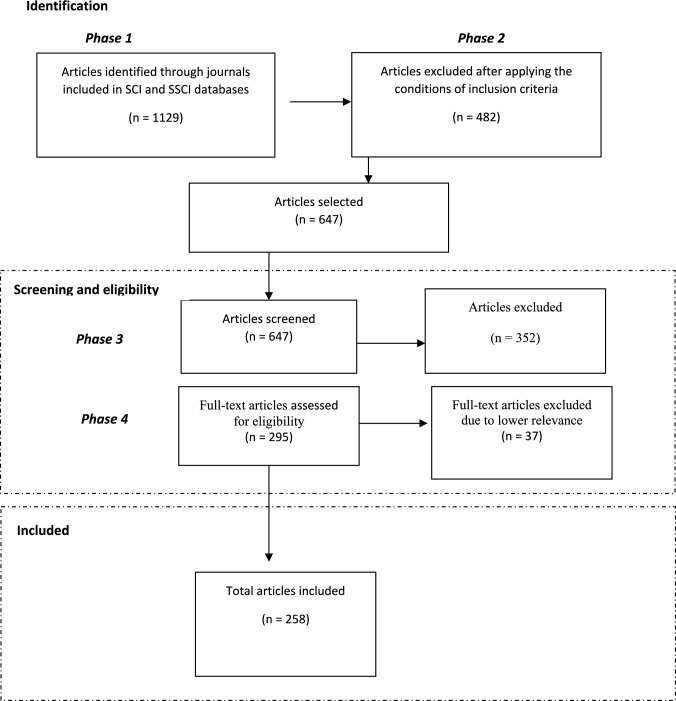
Number of documents identified, included, and excluded based on the search strategy

The establishment of the search strategy and selection of studies for possible inclusion was carried out in four phases (see Fig. 1).

The first phase consisted of selecting a source of bibliographic data. There are several sources for accessing data, e.g. Web of Science (WoS), Scopus, and Google Scholar. For our study, we selected the WoS database by Clarivate Analytics, due to its comprehensive coverage of research on nature-based solutions and climate change, as well as its recognized prestige within the scientific community. However, it was not used for exclusion purposes but only as a starting point, a way to ensure a broad representation of the sound studies on the subject in our sample. Therefore, we selected bibliographic references from journals indexed in the Science Citation Index (SCI) and Social Science Citation Index (SSCI) of the WoS database.

This phase was conducted in two steps. First, we identified the categories of study of SCI and SSCI that fit our subject: nature-based solutions and climate change. We did not impose any restrictions, thereby enabling us to find as many articles as possible. The final selection of categories included Environmental Studies, Environmental Science, Urban Studies, Ecology, Biology, Biodiversity Conservation, Geography, Multidisciplinary, Green and Sustainable Science and Technology, and Water Resources. Second, we selected the keywords. In bibliometric studies, keywords are regarded as fundamental elements for representing the core themes addressed in the papers and illustrate how these themes are interconnected (Callon et al. 1991; Cobo et al. 2011b, Zupic and Čater 2015). The search was carried out in May 2023, and the combination of keywords used was based on previous research (Brink et al. 2016; Cohen-Shacham et al. 2016; Faivre et al. 2017; Chausson et al. 2020; Kumar et al. 2020; Ruangpan et al. 2020; Cousins 2021; Liu et al. 2021; Bunclark and De la Vega Hernández 2022; Turner et al. 2022; Goodwin et al. 2023; Zhong et al. 2023). We conducted a search using the following keywords and their combinations: [(“natural solutions”) OR (“nature-based solutions”) OR (“NBS”)] AND [(“climate”) OR (“adaptation”) OR (“mitigation”) OR (“ecosystem services”) OR (“landscape”) OR (“green infrastructure”) OR (“biodiversity”) OR (“disaster risk”)]. Our advanced query retrieved 1129 initial results from the WoS.

Subsequently, in the second phase, the corpus was further restricted to refine and delimit the search by applying the conditions of the inclusion criteria: publication year (2009–2023 period), document type (articles and reviews), and language (English). This process resulted in a total of 647 papers.

In the third phase, the 647 papers were desk reviewed. To this end, a preliminary content screening was conducted to exclude documents unrelated to the scope of the research. As a result, 352 papers were eliminated either due to duplication or irrelevance to the study’s focus, leaving 295 documents for further analysis. In the process of reading the papers, cited references were used as a secondary source, but this did not yield many additional papers, which can be taken as an indication of the validity of the research.

In the fourth phase, all 295 papers were read in full. This thorough review led to the exclusion of 37 documents that had initially been selected based on their abstracts, due to their limited relevance. As a result, 258 papers from 103 different sources were ultimately selected, covering the period from 2009 to May 2023. The list of 258 papers is provided in the 10.1007/s13280-025-02273-y of the Supplementary Information.

The databanks, journals, and individual papers we used were checked by several researchers to enhance the reliability of the research. It is worth noting that the final selection of papers was carried out independently by the authors, resulting in an intercoder reliability of 93%, indicating a high level of consistency among researchers. Any discrepancies were resolved through consensus in a subsequent working meeting.

### Methods for organizing and analyzing the selected studies

To analyze the selected articles, we first conducted a bibliometric analysis, followed by a literature review based on the findings from that analysis.

#### Bibliometric analysis

Bibliometric methods have two main uses: performance analysis and science mapping or bibliometric mapping (Cobo et al. 2011a). Performance analysis provides an overall profile of publications on a specific topic using selected bibliometric indicators such as temporal evolution, journal and geographic distribution, research areas, etc. Science mapping offers a spatial representation of how disciplines, fields, publications, etc., are related to each other (Cobo et al. 2011a; 2012). Different aspects of the research field can be analyzed, depending on the information used to build a bibliometric map. The aim of science mapping analysis is to discover the conceptual structure and dynamics of scientific fields (Cobo et al. 2011a).

In this study, we conducted bibliometric analyses using the freely available Science Mapping Analysis Tool (SciMAT) software (Cobo et al. 2012). SciMAT software makes it possible to store bibliographic information in a structured manner, including titles, abstracts, keywords, documents, authors, and citations. The software produces performance indicators based on this dataset, making it easier to identify main research themes through science mapping analysis based on co-word bibliographic networks (Cobo et al. 2012). Therefore, SciMAT was chosen because it incorporates a robust methodology based on bibliometric indicators and bibliographic networks (Cobo et al. 2011a).

We carried out the conceptual science mapping analysis using SciMAT, and applying the three-stage approach proposed by Cobo et al. (2011a):

1. *Findings used to identify the research themes *The bibliometric co-word analysis technique was first proposed by Callon et al. (1986) and is one of the most widely used techniques in scientific mapping. Co-word analysis is a tool that examines the co-occurrence of words. According to Callon et al. (1991), co-word analysis is based on the idea that the co-occurrence of keywords describes the content of the documents in a dataset. Two keywords co-occur when they are used together in the description of a document. The higher the co-occurrence of two keywords in documents, the closer their relationship (Chen et al. 2016). Therefore, main research themes are identified by calculating the co-occurrences of keywords, which create a co-occurrence matrix (Callon et al. 1991). Subsequently, the similarities between the items are calculated using the equivalence index (Callon et al. 1991). Then, a clustering algorithm is applied to identify groups of closely associated keywords, which can consequently be considered research themes (Callon et al. 1991; Cobo et al. 2011b). For each research theme, the keywords and their interconnections form a co-word network that represents a thematic cluster. Each cluster is identified by the most relevant keyword associated with that thematic cluster. Its graphical representation is a sphere, whose size is proportional to its importance (Cobo et al. 2011a, 2012). It is important to highlight that thematic clusters are built using keywords, not the papers themselves. Keywords reflect the primary themes covered by the papers and show how they are connected to each other (Callon et al. 1991; Cobo et al. 2011b). Thus, a single article may be related to more than one cluster.
2. *Visualizing research themes and the thematic network *In this stage, the themes are visualized using two different visualization tools: strategic diagrams and thematic networks (Cobo et al. 2011a). Figure 2 is a graphical representation of a two-dimensional strategic diagram based on the density and centrality of the themes. Centrality measures the degree of a network’s interaction with other networks and can be interpreted as an indicator of the strength of external ties with other themes. It can also be understood as a measure of the importance of a theme in the overall development of the research field. Density, on the other hand, measures the strength of internal connections between all the keywords that describe the research theme, and can be understood as an indicator of the theme’s level of development (Callon et al. 1991).

**Fig. 2:**
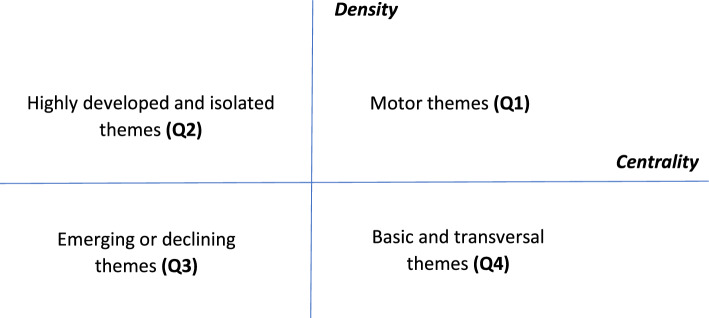
Description of the strategic diagram. *Source* Cobo et al., (2011a, 2012)
  There are four kinds of themes (Callon et al. 1991), classified by the quadrant in which they appear in the strategic diagram (Cobo et al. 2012). The most important and basic themes for structuring a research topic are in the upper-right quadrant (Q1). These themes are known as motor themes, because they present strong centrality and high density. These themes are well-developed and important for the structure of a research field. Themes located in the upper-left quadrant (Q2) are very specialized and possess a peripheral character. These themes are considered to have marginal importance to the field since they have well-developed internal ties but unimportant external ties. Themes located in the lower-left quadrant (Q3) exhibit low density and low centrality, and may represent emerging or disappearing themes, because they are weakly developed and marginal. This quadrant is important for identifying possible emerging themes of future research. Themes located in the lower-right quadrant (Q4) represent transversal, general, and basic topics. These themes are important for the research field but are not well-developed internally.
3. *Results* This stage is where the results from the bibliometric study are presented.

#### Literature review

After identifying the thematic clusters, we conducted a systematic literature review (Tranfield et al. 2003; Jalonen 2012; Sutton et al. 2019) to enrich and complement the findings of the bibliometric analysis. We carried out a comprehensive reading of the papers within each cluster and applied qualitative content analysis through direct identification as the methodological approach (Patton 2002; Mayring 2014). We examined every article in depth and, in line with direct identification, we summarized their objectives, methods, and results in a large table that provided the basis for subsequent analysis and discussion. Two researchers independently coded each article to minimize subjectivity, achieving an intercoder reliability of 89%. We subsequently reached a consensus in a follow-up meeting.

The main findings of the bibliometric analyses are presented in Sects. "Results. This stage is where the results from the bibliometric study are presented." and "Co-word analysis", while Sect. "Literature review" provides a literature review based on the results of the co-word analysis.

## Profile of papers in terms of time, journals, geography, and research areas

The core body of literature we identified comprises 258 papers. This section aims to provide an overall profile of these publications in terms of their distribution over the study period, journal contributions, geographical distribution, and research areas. The bibliometric analysis carried out was descriptive in nature. Consequently, this section provides information to answer the first research question.

### Distribution of publications in the period researched

**Fig. 3:**
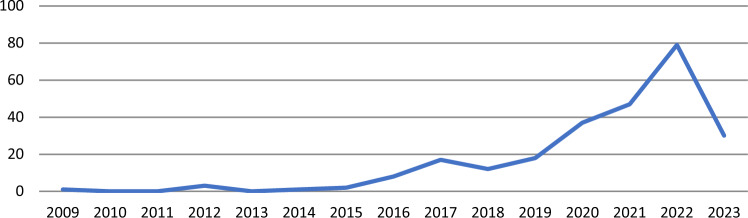
Number of publications per year

The number of publications on a given topic can act as an indicator of scientific activity. The allocation of the publications in the period researched (2009–2023) is presented in Fig. 3.

While 2008 is the first year of publication we took to search for works, the first published papers found were from 2009. The starting point of the study period was chosen to align with the World Bank Report by MacKinnon et al. (2008), in which the concept of nature-based solutions was first introduced. We observed that the evolution of publications in the field appears to follow three distinct stages: an initial stage (2009–2015) with only 9 articles; a pre-expansion stage (2016–2019) with 55 publications; and an expansion stage (2020–2023) showing significant growth in publications, with 194 published articles representing approximately 75% of the dataset. This upward trend in publication volume highlights the growing interest the topic has generated within the scientific community.

### Contribution of scientific journals

The articles included in our sample were published across 103 different journals. The first twenty results are presented in Table 2, in which we included the number of publications and the number of citations. *Environmental Science & Policy* (23 papers), *Science of the Total Environment* (18 papers), *Ambio* (12 papers), *Land Use Policy* (9 papers), and *Environmental Research* (9 papers) published the highest number of papers. Some of these journals have also published special issues on nature-based solutions and climate change, such as *Science of the Total Environment* (Vol. 543, Part B, 2016) and *Ambio* (Vol. 50, Issue 8, 2021).

**Table 2:** List of journals with the highest output and citations

Journal	Publications	Citations
Environmental Science & Policy	23	1324
Science of the Total Environment	18	1337
Ambio	12	134
Land Use Policy	9	437
Environmental Research	9	1225
Urban Forestry & Urban Greening	8	139
Global Change Biology	8	343
Journal of Environmental Management	8	55
Global Environmental Change-Human and Policy Dimensions	6	238
Ecology and Society	6	695
Environmental Research Letter	5	20
Philosophical Transactions of the Royal Society B-Biological Sciences	5	612
Regional Environmental Change	4	21
Climate Policy	4	17
Ecosystem Services	4	184
Global Sustainability	4	61
Landscape and Urban Planning	4	255
Journal of Cleaner Production	3	93
Cities	3	138
Mitigation and Adaptation Strategies for Global Change	3	203

Another way to assess the influence of journals is by examining the number of citations they have received. As shown in Table 2, the journals with the highest number of citations are *Science of the Total Environment* (1337), *Environmental Science & Policy* (1324), *Environmental Research* (1225), and *Ecology and Society* (695). These results reflect the increasing attention this field has garnered among researchers in recent years.

In addition, analyzing the most cited articles within a given discipline offers insights into the academic literature that the research community considers most relevant. Table 3 shows the ten articles that received the most citations. All of these articles are considered to be crucial in the evolution of the field. The article 'Natural climate solutions' by Griscom et al. (2017), published in *Proceedings of The National Academy of Sciences of The United States of America*, received the highest number of total citations (1082). The most significant contribution of the article is the quantification of natural climate solutions to increase carbon storage, with the authors concluding that natural climate solutions could provide 37% of the cost-effective CO_2_ mitigation needed by 2030 for a greater than 66% chance of keeping the temperature below 2 °C. 'Nature-based solutions to climate change mitigation and adaptation in urban areas: Perspectives on indicators, knowledge gaps, barriers, and opportunities for action' by Kabisch et al. (2016) published in *Ecology and Society*, had the next highest number of total citations (487). 'The science, policy and practice of nature-based solutions: An interdisciplinary perspective' by Nesshöver et al. (2017) published in *The Science of the Total Environment,* was the third most cited. The number of citations keeps rising every year.

**Table 3:** List of papers with more citations

Title	Authors	Journal	Year	Total citations
Natural climate solutions	Griscom, B.W; Adams, J; Ellis, PW; Houghton, RA; Lomax, G; Miteva, DA; Schlesinger, WH; Shoch, D; Siikamaki, JV; Smith, P; Woodbury, P; Zganjar, C; Blackman, A; Campari, J; Conant, RT; Delgado, C; Elias, P; Gopalakrishna, T; Hamsik, MR; Herrero, M; Kiesecker, J; Landis, E; Laetadius, L; Leavitt, SM; Minnemeyer, S; Polasky, S; Potapov, P; Putz, FE; Sanderman, J; Silvius, M; Wollenberg, E; Fargione, J	Proceedings of The National Academy of Sciences of The United States of America	2017	1082
Nature-based solutions to climate change mitigation and adaptation in urban areas: Perspectives on indicators, knowledge gaps, barriers, and opportunities for action	Kabisch, N; Frantzeskaki, N; Pauleit, S; Naumann, S; Davis, M; Artmann, M; Haase, D; Knapp, S; Korn, H; Stadler, J; Zaunberger, K; Bonn, A	Ecology and Society	2016	487
The science, policy and practice of nature-based solutions: An interdisciplinary perspective	Nesshover, C; Assmuth, T; Irvine, KN; Rusch, GM; Waylen, KA; Delbaere, B; Haase, D; Jones-Walters, L; Keune, H; Kovacs, E; Krauze, K; Kulvik, M; Rey, F; Van Dijk, J; Vistad, OI; Wilkinson, ME; Wittmer, H	The Science of the Total Environment	2017	462
The superior effect of nature-based solutions in land management for enhancing ecosystem services	Keesstra, S; Nunes, J; Novara, A; Finger, D; Avelar, D; Kalantari, Z; Cerda, A	The Science of the Total Environment	2018	454
A framework for assessing and implementing the co-benefits of nature-based solutions in urban areas	Raymond, CM; Frantzeskaki, N; Kabisch, N; Berry, P; Breil, M; Nita, MR; Geneletti, D; Calfapietra, C	Environmental Science & Policy	2017	402
Urban natural environments as nature-based solutions for improved public health—A systematic review of reviews	van den Bosch, M; Sang, AO	Environmental Research	2017	371
Understanding the value and limits of nature-based solutions to climate change and other global challenges	Seddon, N; Chausson, A; Berry, P; Girardin, CAJ; Smith, A; Turner, B	Philosophical Transactions of the Royal Society B-Biological	2020	299
Marine reserves can mitigate and promote adaptation to climate change	Roberts C.M., O'Leary B.C., Mccauley D.J., Cury P.M., Duarte C.M., Lubchenco J., Pauly D., Sáenz-Arroyo A., Sumaila U.R., Wilson R.W., Worm B., Castilla J.C.,	Proceedings of the National Academy of Sciences of the United States of America	2017	265
Nature-based Solutions: New Influence for Environmental Management and Research in Europe	Eggermont, H; Balian, E; Azevedo, JMN; Beumer, V; Brodin, T; Claudet, J; Fady, B; Grube, M; Keune, H; Lamarque, P; Reuter, K; Smith, M; van Ham, C; Weisser, WW; Le Roux, X	Gaia-Ecological Perspectives for Science and Society	2015	259
Nature-Based Solutions in the EU: Innovating with nature to address social, economic and environmental challenges	Faivre, N; Fritz, M; Freitas, T; de Boissezon, B; Vandewoestijne, S	Environmental Research	2017	241

### Geographical distribution

During the study period, the UK, the USA, Germany, Sweden, and Italy emerged as the leading countries for research on nature-based solutions and climate change. This information is based on the authors' affiliations. Countries with publications were included if at least one author was affiliated with an institution in that country.

## Research areas and Web of Science categories

Table 4 shows the most relevant research areas and Web of Science categories of the papers in our sample. These classifications allow us to identify the disciplines from which knowledge on nature-based solutions and climate change is being produced. The top research areas were Environmental Sciences & Ecology, Water Resources, and Biodiversity & Conservation, while the leading Web of Science categories were Environmental Sciences, Environmental Studies, and Ecology.

**Table 4:** Relevant research areas and WoS categories (2009–2023)

Position	Research areas	Position	WoS categories
1	Environmental Sciences & Ecology	1	Environmental Sciences
2	Water Resources	2	Environmental Studies
3	Biodiversity & Conservation	3	Ecology
4	Urban Studies	4	Water Resources
5	Geology	5	Biodiversity Conservation
6	Plant Sciences	6	Urban Studies
7	Geography	7	Green & Sustainable Science & Technology
8	Life Sciences & Biomedicine	8	Geography
		9	Biology
		10	Multidisciplinary

## Co-word analysis

In this section, the findings of the co-word analysis in the field of nature-based solutions and climate change are explained. Consequently, this section answers the second and third research questions.

As seen in the previous section, performance indicators provide an overall profile of the research field on nature-based solutions and climate change and offer an up-to-date synthesis of the existing literature. However, they do not reveal the structure of the field. To this end, co-word analysis facilitates the development of scientific maps that illustrate how specific research fields are conceptually, intellectually, and socially structured (Cobo et al. 2011a). As stated earlier in the methodology section, these maps offer a spatial representation of how different units of analysis (such as documents, journals, and words) are interrelated through bibliometric networks (Cobo et al. 2012). Co-word analysis uses keywords as units of analysis to detect the themes of a research field and the links between them (Cobo et al. 2011b). Keywords represent the most important themes covered by the papers and how they are linked to each other (Zupic and Čater, 2015). Consequently, one article can be related to more than one cluster.

In this study, we used the original keywords assigned by the authors in their papers and additional relevant keywords assigned by the WoS to each document to obtain a spatial representation of the thematic clusters within the field. A de-duplicating process was applied to improve data quality, by grouping words representing the same concept. Additionally, some meaningless keywords with very broad and general meanings were removed (Cobo et al. 2015). In this way, general keywords such as “nature-based solutions” and “climate”, which were initially used to delimit the dataset, were excluded from the analysis.

**Fig. 4:**
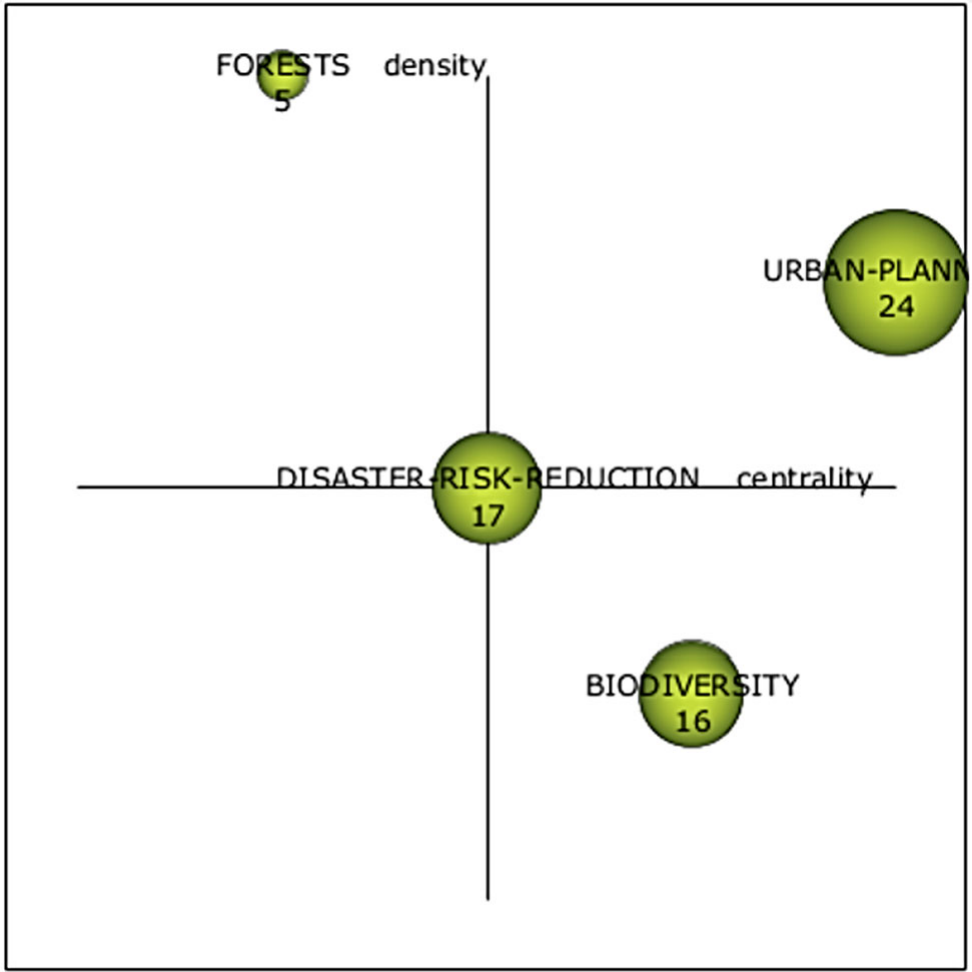
Strategic diagram of nature-based solutions and climate change

The keywords and their interconnections form a co-word network for each research theme, which is visually represented as a thematic cluster (Cobo et al. 2011a). Each cluster is labeled using the most significant keyword in that thematic cluster. Its graphical representation is a sphere, the size of which is proportional to its importance (Cobo et al. 2011a, 2012). After conducting the co-word analysis, the resulting thematic clusters are displayed in the strategic diagram shown in Fig. 4.

This analysis identified four thematic clusters representing the most prominent conceptual cores in the literature on nature-based solutions and climate change. Although the corpus could have generated more subtopics, the level of aggregation adopted made it possible to identify clearly differentiated macro themes, facilitating a strategic interpretation of the field. The clusters identified are: (1) *urban planning*, to ensure the stability and functionality of ecosystems, (2) *disaster risk reduction,* for resilient communities, (3) *forests,* for climate change mitigation, and (4) the role of *biodiversity,* as the foundation of sustainability. Each cluster groups a set of keywords that frequently co-occur in the articles, indicating that they address related aspects within the same thematic line.

As previously mentioned in the methodology section, according to Cobo et al. (2011a), the *urban planning* cluster can be considered a motor theme in nature-based solutions and climate change research, as it is in the upper-right quadrant of the strategic diagram—characterized by a high degree of internal development and strong ties with other concepts within the research field. In general, this motor theme has been the most important in developing the field of nature-based solutions and climate change and has also attracted the greatest focus from researchers. The *forest* theme is in the upper-left quadrant of the strategic diagram. This thematic cluster is not closely related to the other clusters due to its low centrality. This cluster exhibits a high degree of internal development but is only marginally important in relation to the other research fields. The *disaster risk reduction* cluster is located at the center of the strategic diagram, positioned midway between the top-right and bottom-right quadrants, as well as the top-left and bottom-left quadrants. This means that it holds a transitional position within the research field, neither highly developed nor strongly connected, indicating that it may be an emerging research area with potential for further integration and development in future studies. The *biodiversity* theme, located in the lower-right quadrant of the strategic diagram, shows a moderate level of centrality and low density, indicating that while it is connected to other thematic clusters, it remains internally underdeveloped. It is important for the research field as a basic, transversal theme and may provide valuable directions for future research.

**Fig. 5:**
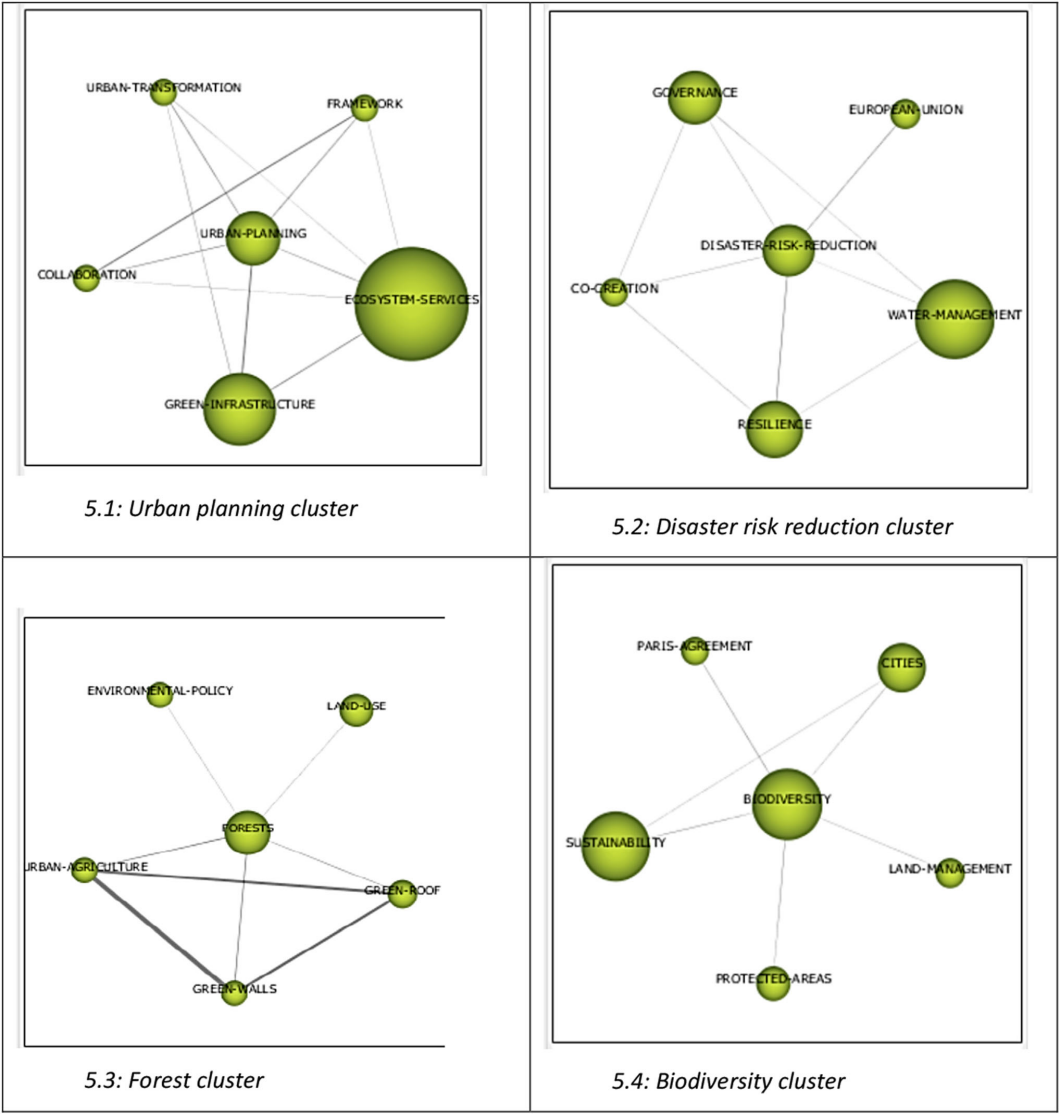
Sub-themes of the thematic clusters

Each theme represented in the strategic diagram is associated with a corresponding keyword network or thematic network, which is illustrated in Fig. 5.

## Literature review

We carried out an in-depth review of each thematic cluster based on the results of the co-word analysis shown in Figs. 4 and 5, with the aim of providing a comprehensive understanding of them. To this end, we conducted a systematic literature review (Tranfield et al. 2003; Jalonen 2012; Sutton et al. 2019) to complement the bibliometric analysis of nature-based solutions and climate change. Consequently, we adopted a qualitative approach at this stage of the study (Patton 2002; Mayring 2014). Specifically, we performed a thorough reading of the papers whose keywords were included in each thematic cluster to identify their objectives, methods, and main contributions.

In short, we were able to interpret and complement the results of the bibliometric analysis through this literature review, gain a deeper understanding of the main findings and provide valuable information on the themes, gaps, and research lines present in each cluster. This analysis contributes to a better understanding of the current conceptual structure of the research field and provides additional insights in response to the third research question on emerging research trends and topics that warrant further attention, thereby identifying potential avenues for future investigation.

### Urban planning cluster

The most important cluster in the strategic diagram (see Fig. 4), located in the upper-right quadrant, is called *Urban planning.* There are several related co-words within this cluster: “urban planning”, “ecosystem services”, “green infrastructure”, “urban transformation”, “collaboration”, and “framework”, as shown in Fig. 5.1. The themes within this cluster are characterized by a high degree of internal development and strong connections with other concepts in the research field.

Ecosystem services are commonly categorized into four categories: supporting services, provisioning services, regulating services, and cultural services (Babi Almenar et al. 2021). Their analysis, particularly the role of nature-based solutions in providing ecosystem services that address climate change and biodiversity loss, has been a predominant theme in the field of research (Brink et al. 2016; Langergraber et al. 2020; Wickenberg et al. 2021; Duffaut et al. 2022; Esperon-Rodriguez et al. 2022). Moreover, ecosystem-based approaches to climate change adaptation are actively promoted at international, national, and local levels by both academics and practitioners (Cohen-Shacham et al. 2016; Kremer et al. 2016; Seddon et al. 2020a).

However, several scholars have highlighted the institutional, political, and socioeconomic barriers that continue to hinder the effective integration of these approaches into urban planning. For instance, Wamsler et al. (2016) identified a lack of inter-institutional coordination and the absence of coherent regulatory frameworks as key obstacles to their implementation. Zölch et al. (2017) highlighted the fragmentation of responsibilities across different levels of government, along with a limited integration of scientific knowledge into decision-making processes, as major constraints to adopting ecosystem-based approaches. Furthermore, Pan et al. (2021) emphasized the importance of strengthening public participation and enhancing the capacity of local actors to overcome the socioeconomic and cultural barriers that hinder the effective implementation of these solutions. Ultimately, local urban planning practices that support these approaches are still fragmented. Measures are often implemented in a piecemeal rather than systematic or comprehensive manner, and they are rarely subjected to thorough evaluation. Therefore, when analyzing the role of nature-based solutions in urban planning, it is important to look beyond the solutions themselves and consider the institutional, governmental, and contextual factors that shape their implementation.

Figure 5.1 illustrates that research within this thematic cluster has also examined the role of green infrastructure in urban planning. Although the concept of green infrastructure is becoming increasingly popular, its definitions, terminology, and objectives vary across geographical and disciplinary contexts (Derkzen et al. 2017; Matsler et al. 2021). Various disciplines (such as urban planning, urban forestry, ecology, engineering, landscape architecture, and law) employ divergent conceptualizations to operationalize the term. Moreover, multiple related terms exist, each associated with distinct academic communities. According to Matsler et al. (2021), these different conceptualizations and terms can be grouped into three main categories: green infrastructure as a green space planning concept, green infrastructure as an urban ecology concept, and green infrastructure as a water and stormwater management concept. However, they also found that many studies fail to clearly define the term, leading to confusion, contradictions, and the ineffective implementation of green infrastructure initiatives. As a result, they argue that scholars and practitioners must be explicit and precise when defining green infrastructure and its purposes in their studies and projects to prevent the siloing of research and practice.

Supporting its practical relevance, Van den Bosch and Sang (2017) noted that green infrastructure can help to tackle problems arising from inappropriate urbanization processes, such as the increase in impervious surfaces that tend to aggravate the consequences of natural disasters. In a similar vein, Anderson and Gough (2020) demonstrated that various applications of green infrastructure in urban, suburban, and peri-urban settings in Ontario (Canada), significantly reduced carbon dioxide concentrations, regardless of land use, location, or geography—highlighting the broad environmental benefits of such strategies. In their analysis of 601 European cities, Marando et al. (2022) showed that widespread implementation of urban green infrastructure, particularly in arid regions and cities with low tree cover, can contribute to reducing the intensity of the urban heat island effect, promote greater urban resilience, and contribute to both climate change adaptation and mitigation.

As noted by Haase et al. (2017) and Turkelboom et al. (2018), although terms such as nature-based solutions, ecosystem services, and green infrastructure have gained significant popularity in recent years within urban sustainability discourse and policy, this popularity does not imply that they are free from limitations and contradictions. These concepts, therefore, require critical examination, as— despite being framed as neutral and apolitical tools intended to deliver benefits for both environmental protection and economic growth (Haase et al. 2017; Kotsila et al. 2021; Goodwin et al. 2023)—they often serve, in practice, to produce and reproduce socio-spatial inequalities and injustices (Haase et al. 2022).

As shown in Fig. 5.1, another topic identified in this thematic cluster, is urban transformations. Several studies argue that strategies aimed at transforming towns and cities by implementing nature-based solutions are having a positive impact in addressing the growing challenges of climate change—such as rising temperatures, heatwaves, extreme precipitation, floods, droughts, and the desertification of surrounding rural areas—(e.g., Kabisch et al. 2016; Faivre et al. 2017; Nesshöver et al. 2017; Raymond et al. 2017; Cohen-Shacham et al. 2019; Babí Almenar et al. 2021; Frantzeskaki and Bush 2021). However, other studies contend that nature-based solutions are not neutral tools; rather, they are discursive instruments and specific urban practices that play a role in the neoliberalization of urban nature (e.g., Haase et al. 2017; Maes and Jacobs 2017; Toxopeus et al. 2020; Cousins 2021; Sekulova et al. 2021; Anguelovski and Corbera 2023). In other words, nature is increasingly seen as being mobilized as a commodified element of urban neoliberal environmentalism. Consequently, so-called “sustainability fixes”, which selectively integrate ecological objectives into urban planning, are reflected in various urban narratives and practices (Pirro and Anguelovski 2017). These scholars argue that future research and practice related to nature-based solutions should adopt a more critical perspective on the term itself and seek to redirect its use in urban planning away from neoliberal agendas toward more emancipatory and globally just socio-ecological futures (Kotsila et al. 2021; Goodwin et al. 2023). Consequently, further research is needed to explore the political, social, environmental, and economic implications of implementing nature-based solutions in urban contexts.

The fifth theme identified in this cluster is collaboration. Several studies emphasize that multi-stakeholder partnerships and coalitions are critical for overcoming siloed approaches and enhancing urban greening practices (Zölch et al. 2017; Frantzeskaki et al. 2020; Mabon and Shih 2021; García Sánchez and Govindarajulu 2023). However, the benefits of public–private collaborations, which often emerge in austerity-driven contexts, are contested. More specifically, it is unclear whether such collaborations inherently support the implementation of nature-based solutions without undermining the public interest or equitable, long-term access to nature (Toxopeus et al. 2020). Urban planning literature further suggests that many new greening strategies are disproportionately implemented in select urban areas, benefiting middle-class, high-income, and racially or ethnically privileged populations, often to the detriment of vulnerable social groups (Haase et al. 2017; Kotsila et al. 2021; Anguelovski and Corbera 2023).

As shown in Fig. 5.1, the last topic identified in this cluster is framework. The implementation of nature-based solutions is inherently complex due to their multifunctionality, the trade-offs between functions, and the diverse temporal and spatial scales involved. Some authors have explored how various frameworks facilitate their implementation to ensure these solutions have a positive impact on towns and cities, aiming to extract and synthesize the key elements and conditions that enable this process (Wickenberg et al. 2021). Others argue that, while urban planning could play a crucial role in supporting the implementation of nature-based solutions, and managing trade-offs, conflicts, and social equity concerns, significant knowledge gaps remain. In particular, there is still uncertainty about how inherently anthropocentric urban planning processes can meaningfully give voice to non-human nature (Bush and Doyon 2019).

From a more critical perspective, Seddon et al. (2021) discussed both the promises and pitfalls of how nature-based solutions are framed and the extent of their current political traction. Similarly, Osaka et al. (2021) reviewed the various ways natural solutions to climate change have been conceptualized, examining the normative and practical implications of this framing. At a surface level, they found that solutions labeled as “natural” were often framed using technical and social evaluation criteria, and generally perceived as more beneficial, cost-effective, mature, and democratic than their artificial counterparts. However, a deeper analysis revealed that this natural framing often obscured the fact that so-called natural solutions can be just as risky, expensive, immature, and technocratic as artificial ones. In the same vein, Woroniecki et al. (2020) emphasized the need to pay closer attention to the epistemological and power dimensions that are often obscured in discussions of nature-based solutions. They argued that nature is not passive or external to human society; instead, it is expressed through frames that embody both knowledge and power within the social contexts where nature-based solutions are implemented. These framings can either limit or enable the capacity of diverse groups to respond to environmental change.

Overall, the literature emphasizes that while nature-based solutions hold great potential in urban planning, their implementation demands greater conceptual clarity, more systematic practices, stronger governance, and greater attention to equity and justice. Building on these insights, the most relevant area for conducting future research within this cluster is to continue investigating the role that ecosystem services (e.g., Wamsler et al. 2016; Pan et al. 2021; Wickenberg et al. 2021; Duffaut et al. 2022) and green infrastructure (e.g., Van den Bosch and Sang 2017; Anderson and Gough; 2020; Kotsila et al. 2021; Matsler et al. 2021; Haase et al. 2022) play in urban planning to address growing societal challenges. This includes a critical examination of how these solutions are integrated into urban planning frameworks, their alignment with broader sustainability agendas, and their potential to reinforce or challenge existing power relations and socio-spatial inequalities. Several authors call for further research in this direction.

### Disaster risk reduction cluster

As shown in Fig. 5.2, the *Disaster Risk reduction* cluster is formed by the co-words: “disaster risk reduction”, “water management” “resilience”, “governance”, “European Union”, and “co-creation”. This thematic cluster is located at the center of the strategic diagram (see Fig. 4), equidistant from both the upper and lower quadrants on the left and right sides. Its central placement indicates that disaster risk reduction occupies a transitional position within the research field, being neither highly developed nor strongly connected. This suggests that the topic may represent an emerging area of study with growth potential that requires further development.

As Schneider et al. (2022) point out, disaster risk reduction involves identifying, assessing, and reducing the factors that generate risks and damages. It further emphasizes the prioritization of strategies designed to reduce exposure and vulnerability through the integration of comprehensive measures for preventing and mitigating extreme events, alongside efforts to enhance resilience (Calliari et al. 2019; Tyllianakis et al. 2022). Consequently, disaster risk reduction constitutes a multidisciplinary field of research, with the literature focusing on various interconnected domains aimed at advancing effective disaster risk management.

In relation to the motor cluster, disaster risk reduction may emerge as a promising area for academic research. However, existing studies on the involvement of urban planning in disaster risk reduction are inconclusive and present mixed findings. Some research indicates that local planning for disaster risk reduction through nature-based solutions remains limited, often constrained by institutional, financial, or technical barriers (Wamsler et al. 2016; Young et al. 2019). Conversely, other studies argue that remarkable efforts have been made to set up legal frameworks for disaster risk reduction in certain regions, such as the European Union (Faivre et al. 2018; Ferreira et al. 2021; Al Sayah et al. 2022; Calliari et al. 2022). These conflicting results may be due to several reasons, such as contextual differences between regions, the methodological diversity of the studies, the level of participation of local stakeholders, and the lack of standardized indicators to assess the impact of nature-based solutions. Therefore, further research is required in this area.

Water management is one of the most important challenges in disaster risk reduction and, therefore, represents a key topic in this cluster. Within this thematic area, several studies have specifically focused on hydrometeorological risks. These hazards, which include floods, storm surges, landslides, droughts, and heat waves, have significantly impacted human societies and ecosystems, with their effects projected to intensify under future climate scenarios (Debele et al. 2019; Rosenberger et al. 2021; Beceiro et al. 2022; Fitobór et al. 2022; Enu et al. 2023). Our review revealed that research on adopting and scaling-up nature-based solutions for mitigating hydrometeorological risks remains limited, even within the European Union (Ruangpan et al. 2020; Muyambo et al. 2022). Furthermore, we observed that disaster policies generally prioritize post-disaster recovery over the proactive prevention of hydrometeorological events (Debele et al. 2019; Ruangpan et al. 2020). Consequently, promoting and implementing proactive hydrometeorological risk management through policies and practices that incorporate comprehensive planning and protective measures is an urgent and imperative need (Kumar et al. 2020; Enu et al. 2023).

Figure 5.2 also highlights resilience as one of the topics within this cluster. Several papers suggest that nature-based solutions may contribute to reducing the risk of disasters and increasing community resilience by providing essential ecosystem services (Kabisch et al. 2017; Mehryar and Surminski 2021). In the same vein, Nesshöver et al. (2017) contend that nature-based solutions provide a means to align environmental and resilience goals, particularly in contexts where resources are limited and short-term needs may compete with long-term priorities. Similarly, Huang et al. (2020) focus on the resilience of areas prone to natural disasters, especially landslides, arguing that comprehensive assessments can improve disaster risk management strategies and strengthen local resilience.

Governance is another prominent theme within this cluster, as illustrated in Fig. 5.2. Several studies emphasize that effective governance—understood as the coordinated engagement and collaboration of all stakeholders, supported by adequate resources tailored to the specific needs of each area—is crucial for achieving meaningful disaster risk reduction (Wamsler and Raggers 2018; Frantzeskaki et al. 2019; Ruangpan et al. 2020; Kiss et al. 2022; Rifai et al. 2022). Some scholars further argue that a key factor for success lies in implementing good governance practices that are grounded in multidisciplinary approaches and transparency (Kabisch et al. 2016; Nesshöver et al. 2017; Cohen-Shacham et al. 2019). Nevertheless, research on governance models specifically tailored to disaster risk reduction remains limited (Zingraff-Hamed et al. 2021), making it one of the topics to be looked into further.

The last group of studies identified in this cluster focuses on co-creation. Some authors argue that more resilient societies are increasingly recognizing the potential of nature-based solutions in addressing disaster risk and climate change (Anderson and Renaud 2021; Badura et al. 2021; Turner et al. 2022). This growing awareness has contributed to greater public acceptance of their implementation in recent years. However, other scholars point out that a lack of public knowledge, awareness and commitment can act as barriers to perceiving the need for implementing such solutions (Anderson and Renaud 2021; Wamsler et al. 2020a, 2020b). As a result, they suggest that the co-creation and co-management of nature-based solution initiatives by all relevant stakeholders could enhance their perception and, consequently, their success (Wamsler et al. 2020a; Anderson et al. 2022). Ultimately, transcending traditional frameworks of cooperation and participation may foster more transformative adaptation to ongoing socio-ecological challenges (Pedersen Zari et al. 2020).

In general, the literature on this cluster indicates that while nature-based solutions are increasingly acknowledged as valuable tools for mitigating climate-related hazards and enhancing resilience, their integration continues to be fragmented due to governance-related challenges, limited proactive planning, and insufficient stakeholder involvement. Future research and practices should prioritize proactive risk management through policies, planning, and protective measures to address these gaps (e.g., Kumar et al. 2020; Enu et al. 2023). Furthermore, the exploration of governance models (e.g., Wamsler and Raggers 2018; Ruangpan et al. 2020; Zingraff-Hamed et al. 2021; Rifai et al. 2022) and the co-creation and co-management of initiatives aimed at disaster risk reduction (e.g., Pedersen Zari et al. 2020; Anderson et al. 2022) stands out as a promising research trend.

Lastly, we observed that many studies in this cluster rely on quantitative research techniques (e.g., Young et al. 2019; Schneider et al. 2022; Tyllianakis et al. 2022). However, we argue that the study of nature-based solutions for disaster risk reduction would benefit from incorporating more qualitative methods, as certain aspects of this field are difficult to capture through quantitative approaches alone.

### Forest cluster

The third cluster, *Forest,* is in the upper-left quadrant of the strategic diagram (see Fig. 4). The co-words that form this thematic cluster are “forests”, “land use”, “environmental policy”, “urban agriculture”, “green walls”, and “green roof” (see Fig. 5.3). As Cobo et al. (2011a) point out, this thematic cluster shows a high degree of internal development but is of marginal importance in relation to the other research fields. These themes are referred to as highly developed and isolated themes. Consequently, research in this cluster has not attracted the interest of many researchers working in the field of nature-based solutions and climate change. However, this does not mean that this research is of a lower quality, merely that journals and scholars have shown less interest in this thematic area.

Forests still cover more than 30% of the planet's land area, despite massive, ongoing losses (FAO, 2022; Tito et al. 2022). Billions of people depend on them for their livelihoods, food, and water (IPBES 2019). As a result, in recent years attention has been focused on protecting and restoring forests. However, some researchers, activists, and planners are concerned that the expansion of forestry as a nature-based solution to mitigate climate change may rely on non-native crops or monoculture farming, which could lead to maladaptation, particularly in a rapidly changing world where biodiversity-based resilience and multifunctional landscapes are critical (Jin et al. 2020; Seddon et al. 2020a; Strauß et al. 2022). However, as Seddon et al. (2021) also point out, a greater concern is that this may divert attention from the urgent need to rapidly phase out fossil fuel use and protect existing native ecosystems and local resource rights.

One of the themes identified in this cluster is land use. Forests are fundamental elements in the configuration and sustainability of land use, as they provide essential ecosystem functions such as hydrological regulation, carbon storage, and biodiversity conservation (Boisvenue et al. 2022; Tito et al. 2022; Wang et al. 2022). Recent literature has highlighted how land use changes driven by agricultural activities, urbanization, or intensive resource exploitation have negatively impacted the ecological integrity of forest systems and global socioeconomic sustainability (Jin et al. 2020; Ma et al. 2022; Roy et al. 2022). In this context, some studies (Keesstra et al. 2018) have shown the potential of nature-based solutions to improve land health and functionality, cope with hydrological risks, and maintain or restore forests. Such solutions not only facilitate the recovery of degraded ecosystem services but also promote more resilient land management practices. However, other studies (Kotsila et al. 2021; Haase et al. 2022) emphasized that many municipalities have leveraged the concept of nature-based solutions, both in urban and rural areas, to revitalize degraded areas and attract new real estate investments, while deeper issues related to uncontrolled land use and urban development persist. Therefore, land use changes and their impacts remain a critical topic requiring further research and development.

This cluster also covers literature on environmental policy, where, as previously mentioned, a growing body of critical research is emerging—particularly focused on nature-based solutions (including forest-based solutions)—examining their design, promise, contradictions, and limitations (Haase et al. 2017, 2022; Cousins 2021; Anguelovski and Corbera 2023), as well as their practical implementation (Cohen-Shacham et al. 2019; Frantzeskaki et al. 2019; Seddon et al. 2019). In this regard, some authors argue that the current understanding of nature-based solutions reflects the conventional approach to environmental policy and management, which upholds a status quo often deemed to be unsustainable and unjust (Maes and Jacobs 2017; Melanidis and Hagerman 2022). Within the forest context, these critiques manifest in the commodification of forest ecosystem services, for example, which may shift conservation priorities toward economic objectives, overlooking social, cultural, and environmental justice aspects related to the communities that depend on these territories. Consequently, the multifunctional role of forests as climate regulators, biodiversity habitats, and sources of local livelihoods runs the risk of being reduced to their value as “measurable” solutions within dominant political frameworks (Seddon et al. 2020a).

The next topic identified in this cluster is urban agriculture, which is closely linked to urban forests through the concept of urban agroforestry. This concept involves integrating trees and shrubs into urban farms, community gardens, and green corridors. Incorporating fruit trees, nitrogen-fixing species, and living hedgerows around these plots, towns and cities can lead to multiple benefits: enhanced ecosystem services, increased biodiversity, higher climate adaptation capacities, and the promotion of social cohesion and environmental justice (Calliari et al. 2022). Within this framework, Nassary et al. (2022) showed that the development of urban agriculture and arboriculture in the Global South yields interconnected benefits, including better access to food and income generation, enhanced leisure and social interactions, increased biodiversity, and mitigation of environmental pollution through lower greenhouse gas emissions and carbon sequestration. In short, the development of urban agroforestry in urban landscapes represents a promising avenue for future research.

Green roofs and green walls are other themes covered in this cluster. These urban structures integrate vegetation into cityscapes where natural ground surfaces are limited or entirely built over. They are closely related to forests due to their shared environmental benefits and ecological functions. Both forests and green roofs and walls are components of nature-based solutions aimed at enhancing environmental quality, with green roofs and walls serving as urban extensions of the ecological services provided by forests in natural settings. They provide similar ecological benefits in their respective contexts, including better air quality, lower noise pollution, mitigation of climate change and the urban heat island effect, energy savings, and stormwater management (Koch et al. 2020; Liu et al. 2021; Boisvenue et al. 2022; Cortinovis et al. 2022; Su et al. 2022; Twohig et al. 2022).

All in all, this cluster shows that, although forests are widely recognized as fundamental nature-based solutions for climate change mitigation and adaptation, their implementation faces risks of maladaptation, commodification, and social injustice. Land use change remains a critical challenge, posing both significant threats and potential opportunities for ecological restoration. Therefore, future research on this cluster should focus on deepening the understanding of these issues. In particular, the impacts of reforestation policies and land use changes on the rights of local communities, in both rural and urban settings, are emerging as key research trends (e.g., Keesstra et al. 2018; Jin et al. 2020; Kotsila et al. 2021; Haase et al. 2022; Ma et al. 2022). Moreover, the increasing volume of critical literature on environmental policy (e.g., Maes and Jacobs 2017; Cousins 2021; Haase et al. 2022; Melanidis and Hagerman 2022; Anguelovski and Corbera 2023) indicates that a significant amount of current research is focusing on issues related to inequality of benefits and social justice.

### Biodiversity cluster

The *Biodiversity* cluster can be found in the lower-right quadrant (see Fig. 4). Themes located in this quadrant are transversal, general, and basic to the research field, yet they remain underdeveloped in terms of internal structure. As Fig. 5.4 shows, this thematic cluster is formed by the co-words: “biodiversity” “sustainability”, “cities”, “protected areas”, “land management”, and “Paris Agreement”.

Biodiversity encompasses the variety of life on Earth and is essential to maintaining ecological balance. It supports food and medicine production, scientific advancement, and the preservation of cultural values. However, despite the fundamental importance of natural resources, and ongoing efforts to conserve and restore them (Pettorelli et al. 2021), degradation continues, resulting in detrimental impacts on both biodiversity and human well-being (Griscom et al. 2017; Nesshöver et al. 2017; Cohen-Shacham et al. 2019; Mackinnon et al. 2020; Baldwin-Cantello et al. 2023). This continued decline highlights the urgent need to prevent further ecological deterioration (Seddon et al. 2020a).

The relationship between biodiversity and sustainability is addressed in this thematic cluster. Biodiversity plays a key role in sustainability by strengthening the resilience of ecosystems to climate change, as diverse ecological systems are better able to adapt to and maintain their functions in the face of climatic disturbances (IPBES 2019; Malhi et al. 2020). However, as noted by Shin et al. (2022), despite the close links between biodiversity conservation actions and climate change mitigation, these links are seldom as integrated as they should be in management and policy related to them. Even in the context of nature-based solutions, which consider ecosystems to be crucial to mitigating and adapting to the impacts of climate change, the potential role of biodiversity has received little attention (Mori 2020; Roberts et al. 2020). In the same vein, Baldwin-Cantello et al. (2023) pointed out that although policy goals on climate change, biodiversity, and human well-being have been set at local, national, and international levels, the measures implemented so far are insufficient to meet these goals. Therefore, reconsidering and emphasizing the links between nature protection policies and climate change mitigation and adaptation is urgently needed to ensure that collective efforts to improve the environment and human well-being are truly effective.

Research on the impact of climate change and loss of biodiversity in cities has become increasingly important in recent years (e.g., Hobbie and Grimm 2020; Duffaut et al. 2022; Xie et al. 2022). Urban ecosystems present complex challenges, including various forms of pollution, high temperatures compared to surrounding areas, and risks of flooding due to soil sealing. In this context, some authors (Duffaut et al. 2022) argue that nature-based solutions offer a promising approach to address these issues, while simultaneously providing benefits for mitigating climate change and the biodiversity crisis. However, other scholars contend that designing, implementing, evaluating, and improving fair and resilient nature-based solutions capable of tackling the urgent challenges of biodiversity loss and climate change will not be feasible without clear definitions, broad participation, and consensus between all stakeholders regarding their principles, scope, distribution, and practical actions (Chausson et al. 2020; Cousins 2021; Kotsila et al. 2021; Seddon et al 2021; Sekulova et al. 2021). Therefore, further research is required in this direction.

This cluster also includes literature on protected areas. Protected areas have become the cornerstone for biodiversity conservation (Mackinnon et al. 2020; Riisager-Simonsen et al. 2022), although the land area they cover is currently insufficient to fully represent global biodiversity, with many ecosystems being poorly represented in protected area networks (Mackinnon et al. 2020; Roberts et al. 2020; O’Brien et al. 2023). Therefore, traditional reserve systems need to be expanded and it is essential that other novel conservation measures, such as rewilding, which is aimed at restoring ecosystems on a large scale by recovering the functions and relationships between species, are recognized and supported to ensure that biodiversity is conserved effectively (Pasimeni et al. 2019; Carrol and Ray, 2021; Queirós et al. 2021; Gordon et al. 2022).

Land management is another topic covered in this cluster. Land management plays a crucial role in maintaining the delicate balance between meeting growing human needs, protecting and conserving biodiversity, and addressing climate change (IPBES 2019). Therefore, it seems essential to adopt a holistic approach that integrates sustainable land management practices, even though some of these practices may be difficult to implement (Malhi et al. 2020; Baldwin-Cantello et al. 2023).

The last issue addressed in this cluster is the Paris Agreement. Ecosystems around the world and the communities that depend on them are vulnerable to the effects of climate change. However, ecosystems can also protect people from the effects of climate change (Malhi et al. 2020). In this regard, Mori (2020) emphasized that biodiversity should be considered not only as a consequence but also as a key cause in helping society and nature face the challenges associated with climate change. Shin et al. (2022) contended that conservation actions which halt, slow or reverse biodiversity loss can simultaneously slow anthropogenic mediated climate change significantly. For this reason, the signatory countries of the Paris Agreement (United Nations, 2015) expressed their intention to implement nature-based solutions to adapt to climate change.

However, these intentions vary depending on the level of economic development, region, and habitat type, and have very rarely translated into measurable evidence-based targets (Seddon et al. 2020a, b). Despite this, Seddon et al. (2020a, b) went on to argue that nature-based solutions can be crucial in achieving global climate and biodiversity goals, and they urged researchers to work more closely with policymakers to identify targets that simultaneously benefit people, ecosystems, and the climate. In the same vein, Griscom et al. (2017) and Ortiz et al. (2021) pointed out that biodiversity loss and climate change should be considered a deeply interconnected anthropogenic global crisis, with nature-based solutions being, in their view, the appropriate tools to tackle these crises and meet the objectives of the Paris Agreement. By contrast, Strauß et al. (2022) argued that nature-based solutions have proven to be a missed opportunity to tackle the two crises in tandem.

Generally speaking, the papers that make up this cluster, located in the lower-right quadrant, are considered important to this field of research but are not yet well-developed internally (Callon et al. 1991; Cobo et al. 2012). While nature-based solutions offer promising approaches, the literature indicates that their design, implementation, and evaluation often lack clear definitions, stakeholder consensus, and measurable targets. Policy frameworks, such as the Paris Agreement, acknowledge these links, but commitments rarely translate into effective action. Therefore, we recommend directing further research and policy efforts (e.g., Mori 2020; Roberts et al. 2020; Shin et al. 2022) toward jointly addressing climate and biodiversity goals.

## Concluding remarks and directions of future research

This study is a review of the research on nature-based solutions and climate change by combining bibliometric analysis with a literature review. The systematized research protocol, combined with the use of different analysis techniques, provides a solid methodological contribution. Thus, this work provides a comprehensive overview of the development of the research field, identifies the main research themes, and highlights potential directions for future research.

The findings that can be drawn from our study are as follows. First, nature-based solutions to tackle climate change have emerged as a relatively new area of research in the sustainability literature in recent years. In the Web of Science, the first paper on this topic was published in 2009. Since then, the number of publications has steadily increased. The final four years of our analysis (2020–2023) were the most productive, indicating a growing interest in this topic among researchers. Consequently, considering the field's upward trajectory, we expect that the number of publications in future will continue to increase.

Second, the overall profile of the articles also revealed that many journals have published different research topics on nature-based solutions and climate change. As a result, the total number of articles (258) were published across 103 different journals, highlighting the high dispersion of the research. This dispersion in scientific literature is usually common in relatively new research fields. In the first few years we looked at, studies were published in journals that promote research at the intersection of environmental science, policy, society and technology aimed at tackling the challenges of climate change (e.g., *Mitigation and Adaptation Strategies for Global Change*), in specialized journals on social-ecological systems research and resilience (e.g., *Ecology and Society*), and in important environmental research journals (e.g., *Environmental Research*). In recent years, there has been an increase in the number of papers published in journals specializing in environmental science, policy, and society (e.g., *Environmental Science and Policy*) and in journals that focus on the environment and its relationship with humans (e.g., *Science of the Total Environment*). It is also significant that some of the most productive journals devoted special issues to the topic, e.g., *Science of the Total Environment* in 2016 and *Ambio* in 2021.

Third, the co-word analysis identified four thematic clusters: *urban planning, disaster risk reduction, forest, and biodiversity*. Of these, the *urban planning* cluster has been particularly important in terms of the evolution of the field and represents topics that have led the research on nature-based solutions and climate change. While much of the research has focused on analyzing the role that ecosystem services and green infrastructure play in urban planning to tackle growing societal challenges, our review shows that in practice these measures are not applied systematically or comprehensively and have not been reviewed very thoroughly.

Furthermore, institutional, governmental, and contextual factors that enable or hinder the effective integration of these measures into urban planning should also be examined. Although nature-based solutions and green infrastructure are often presented as neutral and apolitical concepts aimed at providing benefits for both environmental conservation and economic development, in practice, they frequently create and reinforce social and spatial inequalities and injustices. In this regard, we agree with several authors (e.g., Haase et al. 2017; Kotsila et al. 2021; Nassary et al. 2022; Goodwin et al. 2023), who call for more research and practical implementation in this direction to ensure that the nature-town/city relationship is formulated in terms of more just and equitable towns and cities.

The *disaster risk reduction* thematic cluster is located at the center of the strategic diagram, meaning that it is made up of themes with internal and external links at less developed levels than in the motor cluster. In relation to the latter, although part of the research has focused on analyzing the involvement of urban planning in disaster risk reduction, findings remain inconclusive and present mixed results, highlighting the need for further investigation to clarify the role and effectiveness of urban planning in this context. It is also necessary to examine why disaster management policies tend to prioritize post-disaster recovery over proactive risk prevention (e.g., Debele et al. 2019; Ruangpan et al. 2020). This reactive approach may be related to the lack of governance models specifically designed for disaster risk reduction, an aspect that has received very little attention in academic literature (e.g., Zingraff-Hamed et al. 2021). Therefore, we believe that greater institutional and political commitment is required, together with better planning and more research on governance models for nature-based solutions to cope with disaster risk.

In the strategic diagram, the *forest* cluster is in the upper-left quadrant, which means that its themes are currently peripheral or of marginal importance to the development of the field. However, our review reveals that the impacts of reforestation policies, land use changes (e.g., Jin et al. 2020; Ma et al. 2022) and uncontrolled urban development (e.g., Kotsila et al. 2021; Haase et al. 2022) are critical topics that have received very little attention. We believe that further research on these topics may help us get a better understanding of their potential in relation to climate change mitigation and adaptation. Moreover, our review highlights the need to re-examine the relationship between environmental policy and neoclassical economic thinking (e.g., Maes and Jacobs 2017; Melanidis and Hagerman 2022; Anguelovski and Corbera 2023) in order to address issues related to the unequal distribution of benefits and social justice.

The *biodiversity* cluster is in the lower-right quadrant of the strategic diagram, meaning that it is made up of basic and transversal themes. These themes are important to the research field but are not yet well-developed internally, so they may represent valuable opportunities for future research. In recent years, there has been a continuous degradation of natural resources that has negatively affected both biodiversity and human well-being. In this context, some authors (e.g., Griscom et al. 2017; Ortiz et al. 2021; Duffaut et al. 2022) argue that nature-based solutions could be a tool to reverse this trend. However, other authors (e.g., Shin et al. 2022) point out that nature-based solutions rarely consider tackling biodiversity loss and climate change mitigation in tandem. Consequently, there is significant research potential in understanding how just and resilient nature-based solutions that tackle both the challenges of climate change and biodiversity loss in tandem can be designed and implemented in national and urban agendas. Adopting a sustainable land management approach could also contribute to better understanding and shape the role of nature-based solutions in meeting global climate and biodiversity goals.

Future research could also look further into assessing the effectiveness of nature-based solutions, especially at large spatial scales, since some authors (e.g., Manes et al. 2023) argue that the potential of such solutions to deliver the expected benefits has not been rigorously evaluated and that doubts remain about their reliability and cost-effectiveness compared to alternatives, as well as their resilience to climate change. In this regard, authors such as Seddon et al. (2020b) and Seddon et al. (2021) note that national intentions to provide nature-based solutions for climate change adaptation vary by level of economic development, region and habitat type, and are rarely translated into measurable evidence-based targets. Nevertheless, Seddon et al. (2020b) also argue that nature-based solutions can play a fundamental role in achieving climate and biodiversity goals. To this end, they advocate for closer collaboration between researchers and policymakers to define targets that generate benefits for people, ecosystems, and the climate.

Overall, our review shows that the existing literature on nature-based solutions and climate change is predominantly empirical. Researchers have employed a wide range of methodological approaches, including both qualitative and quantitative techniques. A significant number of studies adopt mixed-methods research designs, integrating these approaches within a single investigation. This methodological diversity reflects the inherent complexity of the topic and the need to capture both measurable outcomes and context-specific factors, such as stakeholder perceptions, governance dynamics, and social equity considerations.

Longitudinal studies would allow for a deeper, more nuanced understanding of this field of research over time, by facilitating the analysis of changes, impacts, and dynamics that develop progressively in specific contexts. Moreover, they would contribute to assessing the effectiveness of nature-based solutions across various temporal scales and time horizons.

The contributions of this study should be considered in light of several limitations. First, we selected the WoS database as our source of information, as it is an international platform that covers a broad range of academic topics. However, other international databases, such as Scopus and Google Scholar, could have provided additional relevant data that were not considered in our study. Furthermore, some of the earliest studies on this topic were institutional documents, which are not indexed in the WoS database and, therefore, were not included in our analysis. Second, it is worth noting that certain terms included in the search string, such as “biodiversity” and “disaster risk”, emerged as thematic clusters in the co-occurrence maps. This could be interpreted as a direct consequence of the search design. However, it also reflects the centrality of these topics in the field under study. Their inclusion responded to the need to capture relevant literature that, although conceptually aligned with nature-based solutions in climate-related contexts, does not necessarily employ this specific terminology. Nevertheless, we acknowledge that this design may have influenced the formation of certain clusters. Third, a methodological bias may have been introduced during the process of normalizing the keywords, as terms with the same or similar meanings were grouped together. Fourth, we acknowledge that the interpretation of the themes included in each cluster may not fully capture the richness of each theme.

Despite these limitations, we believe that this study provides a comprehensive overview of the current state of research on nature-based solutions and climate change. We consider that the interpretation of the themes included in each cluster constitutes a global organization and a general systematization of the research field’s conceptual structure. The research gaps identified and directions for future research suggested highlight the growing interest in this topic.

## Supplementary Information

Below is the link to the electronic supplementary material.

Supplementary file1 (PDF 389 kb)
