# Supplementary material for: Bibliometric and literature review of research on nature-based solutions and climate change: Implications for policy and practice
Source: Ambio. 2025 Oct 29;55(4):689–713. doi: 10.1007/s13280-025-02273-y (PMC12960867; doi:10.1007/s13280-025-02273-y)
Supplement: Supplementary file 1 — Supplementary file1 (PDF 389 kb) [file 13280_2025_2273_MOESM1_ESM.pdf]

Supplementary Information

Journal: Ambio

Article title: Bibliometric and literature review of research on nature-based solutions and climate change: Implications for policy and practice

Authors: Carmen Echebarria, Izaskun Gomez de Salazar

Email carmen.etxebarria@ehu.eus

Appendix

Articles included in the dataset for conducting the review

Adu Boateng, E., Asibey, M. O., Cobbinah, P. B., Adutwum, I. O., & Blija, D. K. (2023). Enabling nature-based solutions: innovating urban climate resilience. *Journal of Environmental Management*, *332*, 117433–117433. https://doi.org/10.1016/j.jenvman.2023.117433

Agarwal, D. S., & Bharat, A. (2023). Nature-based solutions for flood–drought mitigation using a composite framework: a case-based approach. *Journal of Water and Climate Change*, *14*(3), 778–795. https://doi.org/10.2166/wcc.2023.369

Al Sayah, M. J., Versini, P. A., & Schertzer, D. (2022). H2020 projects and EU research needs for nature-based adaptation solutions. *Urban Climate*, *44*, 101229. https://doi.org/10.1016/j.uclim.2022.101229

Albert, C., Hack, J., Schmidt, S., & Schröter, B. (2021a). Planning and governing nature-based solutions in river landscapes: Concepts, cases, and insights. *Ambio*, 50(8), 1405-1413. https://doi.org/10.1007/s13280-021-01569-z

Albert, C., Brillinger, M., Guerrero, P., Gottwald, S., Henze, J., Schmidt, S., Ott, E., & Schröter Barbara. (2021b). Planning nature-based solutions: principles, steps, and insights. *Ambio*, 50(8), 1446–1461. https://doi.org/10.1007/s13280-020-01365-1

Alves, A., Vojinovic, Z., Kapelan, Z., Sanchez, A., & Gersonius, B. (2020). Exploring trade-offs among the multiple benefits of green-blue-grey infrastructure for urban flood mitigation. *Science of the Total Environment*, 703. https://doi.org/10.1016/j.scitotenv.2019.134980

Anderson, V., & Gough, W. A. (2020). Evaluating the potential of nature-based solutions to reduce ozone, nitrogen dioxide, and carbon dioxide through a multi-type green infrastructure study in Ontario, Canada. *City and Environment Interactions* 6. https://doi.org/10.1016/j.cacint.2020.100043

Anderson, V., & Gough, W. A. (2022). Nature-based cooling potential: a multi-type green infrastructure evaluation in Toronto, Ontario, Canada. *International Journal of Biometeorology*, 66(2), 397–410. https://doi.org/10.1007/s00484-021-02100-5

Anderson, C. C., & Renaud, F. G. (2021). A review of public acceptance of nature-based solutions: the ‘why’, ‘when’, and ‘how’ of success for disaster risk reduction measures. *Ambio*, 50(8), 1552–1573. https://doi.org/10.1007/s13280-021-01502-4

Anderson, C. C., Renaud, F. G., Hanscomb, S., & Gonzalez-Ollauri, A. (2022). Green, hybrid, or grey disaster risk reduction measures: What shapes public preferences for nature-based solutions? *Journal of Environmental Management*, 310, 114727. https://doi.org/10.1016/j.jenvman.2022.114727

Anguelovski, I., & Corbera, E. (2023). Integrating justice in nature-based solutions to avoid nature-enabled dispossession. *Ambio*, *52*(1), 45–53. https://doi.org/10.1007/s13280-022-01771-7

Aquije, C., Schmidt, H.-P., Draper, K., Joseph, S., & Ladd, B. (2021). Low tech biochar production could be a highly effective nature-based solution for climate change mitigation in the developing world. *Plant and Soil: An International Journal on Plant-Soil Relationships*, 479(1-2), 77–83. https://doi.org/10.1007/s11104-021-05159-6

Asif, Z., Chen, Z., Sadiq, R., & Zhu, Y. (2023). Climate change impacts on water resources and sustainable water management strategies in North America. *Water Resources Management*, 37, 2771-2786. https://doi.org/10.1007/s11269-023-03474-4

Aurelle, D., Thomas, S., Albert, C., Bally, M., Bondeau, A., Boudouresque, C. F., ... & Fady, B. (2022). Biodiversity, climate change, and adaptation in the Mediterranean. *Ecosphere*, 13, e3915. https://doi.org/10.1002/ecs2.3915

Babi Almenar J., Rugani, B., Geneletti, D., & Brewer, T. (2018). Integration of ecosystem services into a conceptual spatial planning framework based on a landscape ecology perspective. *Landscape Ecology*, 33(12), 2047–2059. https://doi.org/10.1007/s10980-018-0727-8

Babí Almenar Javier, Elliot, T., Rugani, B., Philippe Bodénan, Navarrete Gutierrez, T., Sonnemann, G., & Geneletti, D. (2021). Nexus between nature-based solutions, ecosystem services and urban challenges. *Land Use Policy*, 100, 104898. https://doi.org/10.1016/j.landusepol.2020.104898

Badura, T., Krkoška Lorencová, E., Ferrini, S., & Vačkářová, D. (2021). Public support for urban climate adaptation policy through nature-based solutions in Prague. *Landscape and Urban Planning,* 215, 104215. https://doi.org/10.1016/j.landurbplan.2021.104215

Baldwin-Cantello, W., Tickner, D., Wright, M., Clark, M., Cornelius, S., Ellis, K., Francis, A., Ghazoul, J., Gordon, J.E., Matthews, N., Milner-Gulland, E.J., Smith, P., Walmsley, S. & Young, L. (2023). The Triple Challenge: synergies, trade-offs and integrated responses for climate, biodiversity, and human wellbeing goals. *Climate Policy*, 23(6), 782-799. https://doi.org/10.1080/14693062.2023.2175637

Bastian, O., Haase, D., & Grunewald, K. (2012). Ecosystem properties, potentials and services–The EPPS conceptual framework and an urban application example. *Ecological Indicators*, *21*, 7-16. https://doi.org/10.1016/j.ecolind.2011.03.014

Beceiro, P., Brito, R. S., & Galvão, A. (2022). Nature-based solutions for water management: insights to assess the contribution to urban resilience. *Blue-Green Systems*, *4*(2), 108-134. https://doi.org/10.2166/bgs.2022.009

Bennett, G., Cassin, J., & Carroll, N. (2016). Natural infrastructure investment and implications for the nexus: a global overview. *Ecosystem Services*, 17, 293-297. https://doi.org/10.1016/j.ecoser.2015.05.006

Biswal, B. K., Bolan, N., Zhu, Y.-G., & Balasubramanian, R. (2022). Nature-based systems (NbS) for mitigation of stormwater and air pollution in urban areas: a review. *Resources, Conservation & Recycling*, 186. https://doi.org/10.1016/j.resconrec.2022.106578

Blackwood, L., & Renaud, F. G. (2022). Barriers and tools for implementing nature-based solutions for rail climate change adaptation. *Transportation Research Part D*, *113*.https://doi.org/10.1016/j.trd.2022.103529

Boisvenue, C., Paradis, G., Eddy, I. M. S., McIntire, E. J. B., & Chubaty, A. M. (2022). Managing forest carbon and landscape capacities. *Environmental Research Letters*, 17(11). https://doi.org/10.1088/1748-9326/ac9919

Botero-Acosta, A., Ficklin, D. L., Ehsani, N., & Knouft, J. H. (2022). Climate induced changes in streamflow and water temperature in basins across the Atlantic Coast of the United States: An opportunity for nature-based regional management. *Journal of Hydrology: Regional Studies*, 44, 101202. https://doi.org/10.1016/j.ejrh.2022.101202

Bradfer-Lawrence, T., Finch, T., Bradbury, R. B., Buchanan, G. M., Midgley, A., & Field, R. H. (2021). The potential contribution of terrestrial nature-based solutions to a national 'net zero' climate target. *Journal of Applied Ecology*, 58(11), 2349–2360. https://doi.org/10.1111/1365-2664.14003

Brink, E., Aalders, T., Ádám, D., Feller, R., Henselek, Y., Hoffmann, A., Ibe, K., Matthey-Doret, A., Meyer, M., Negrut, N. L., Rau, A.-L., Riewerts, B., von Schuckmann, L., Törnros, S., von Wehrden, H., Abson, D. J., & Wamsler, C. (2016). Cascades of green: a review of ecosystem-based adaptation in urban areas. *Global Environmental Change*, 36, 111–123. https://doi.org/10.1016/j.gloenvcha.2015.11.003

Brink, E., & Wamsler, C. (2018). Collaborative governance for climate change adaptation: mapping citizen-municipality interactions. *Environmental Policy and Governance*, 28(2), 82–97. https://doi.org/10.1002/eet.1795

Bunclark, L., & De la Vega Hernández, I. M. (2022). Scientific mapping of research on nature-based solutions for sustainable water management. *Water Resources Management*, *36*(12), 4499–4516.https://doi.org/10.1007/s11269-022-03242-w

Bush, J., & Doyon, A. (2019). Building urban resilience with nature-based solutions: how can urban planning contribute? *Cities*, 95, 102483. https://doi.org/10.1016/j.cities.2019.102483

Bustamante, M. M. C., Silva José Salomão, Scariot, A., Sampaio, A. B., Mascia, D. L., Garcia, E., Sano, E., Fernandes, G. W., Durigan, G., Roitman, I., Figueiredo, I., Rodrigues, R. R., Pillar Valério D, de Oliveira, A. O., Malhado, A. C., Alencar, A., Vendramini, A., Padovezi Aurélio, Carrascosa, H., … Nobre, C. (2019). Ecological restoration as a strategy for mitigating and adapting to climate change: lessons and challenges from Brazil. *Mitigation and Adaptation Strategies for Global Change*, 24(7), 1249–1270. https://doi.org/10.1007/s11027-018-9837-5

Caldecott, J. O. (2022). Implications of earth system tipping pathways for climate change mitigation investment. *Discover Sustainability*, *3*(1), 37. https://doi.org/10.1007/s43621-022-00105-7

Calliari, E., Staccione, A., & Mysiak, J. (2019). An assessment framework for climate-proof nature-based solutions. *Science of the Total Environment*, *656*, 691–700. https://doi.org/10.1016/j.scitotenv.2018.11.341

Calliari, E., Castellari, S., Davis, M. K., Linnerooth-Bayer, J., Martin, J., Mysiak, J., Pastor, T., Ramieri, E., Scolobig, A., Sterk, M., Veerkamp, C., Wendling, L., & Zandersen, M. (2022). Building climate resilience through nature-based solutions in Europe: a review of enabling knowledge, finance and governance frameworks. *Climate Risk Management*, 37. https://doi.org/10.1016/j.crm.2022.100450

Carroll, C., & Ray, J. C. (2021). Maximizing the effectiveness of national commitments to protected area expansion for conserving biodiversity and ecosystem carbon under climate change. *Global Change Biology*, 27(15), 3395–3414. https://doi.org/10.1111/gcb.15645

Castellar, J. A. C., Popartan, L. A., Pueyo-Ros, J., Atanasova, N., Langergraber, G., Säumel, I., Corominas, L., Comas, J., & Acuña, V. (2021). Nature-based solutions in the urban context: terminology, classification and scoring for urban challenges and ecosystem services. *Science of the Total Environment*, 779, 146237. https://doi.org/10.1016/j.scitotenv.2021.146237

Chan, F. K. S., Griffiths, J. A., Higgitt, D., Xu, S., Zhu, F., Tang, Y.-T., Xu, Y., & Thorne, C. R. (2018). "Sponge City" in China—a breakthrough of planning and flood risk management in the urban context. *Land Use Policy*, *76*, 772–778.https://doi.org/10.1016/j.landusepol.2018.03.005

Chatzimentor, A., Apostolopoulou, E., & Mazaris, A. D. (2020). A review of green infrastructure research in Europe: challenges and opportunities. *Landscape and Urban Planning*, 198, 103775. https://doi.org/10.1016/j.landurbplan.2020.103775

Chausson, A., Turner, B., Seddon, D., Chabaneix, N., Girardin, C. A. J., Kapos, V., Key, I., Roe, D., Smith, A., Woroniecki, S., & Seddon, N. (2020). Mapping the effectiveness of nature-based solutions for climate change adaptation. *Global Change Biology*, *26*(11), 6134–6155.https://doi.org/10.1111/gcb.15310

Cheng, Z., Nitoslawski, S., Konijnendijk van den Bosch, C., Sheppard, S., Nesbitt, L., & Girling, C. (2021). Alignment of municipal climate change and urban forestry policies: a Canadian perspective. *Environmental Science and Policy*, 122, 14–24. https://doi.org/10.1016/j.envsci.2021.04.005

Cheng, S.H., Costedoat, S., Sterling, E.J. et al. (2022). What evidence exists on the links between natural climate solutions and climate change mitigation outcomes in subtropical and tropical terrestrial regions? A systematic map protocol. *Environmental Evidence* 11(1), 15. https://doi.org/10.1186/s13750-022-00268-w

Choi, C., Berry, P., & Smith, A. (2021). The climate benefits, co-benefits, and trade-offs of green infrastructure: a systematic literature review. *Journal of Environmental Management*, *291*, 112583. https://doi.org/10.1016/j.jenvman.2021.112583

Cohen-Shacham, E., Andrade, A., Dalton, J., Dudley, N., Jones, M., Kumar, C., Maginnis, S., Maynard, S., Nelson, C. R., Renaud, F. G., Welling, R., & Walters, G. (2019). Core principles for successfully implementing and upscaling nature-based solutions. *Environmental Science and Policy*, *98*, 20–29.https://doi.org/10.1016/j.envsci.2019.04.014

Connop, S., Vandergert, P., Eisenberg, B., Collier, M. J., Nash, C., Clough, J., & Newport, D. (2016). Renaturing cities using a regionally-focused biodiversity-led multifunctional benefits

approach to urban green infrastructure. *Environmental Science and Policy*, 62, 99–111. https://doi.org/10.1016/j.envsci.2016.01.013

Cortinovis, C., Olsson, P., Boke-Olén, N., & Hedlund, K. (2022). Scaling up nature-based solutions for climate-change adaptation: potential and benefits in three European cities. *Urban Forestry & Urban Greening*, 67, 127450. https://doi.org/10.1016/j.ufug.2021.127450

Costa, M. M., Marchal, R., Moncoulon, D., & Martín, E. G. (2020). A sustainable flywheel: Opportunities from insurance'business to support nature-based solutions for climate adaptation. *Environmental Research Letters*, 15(11). https://doi.org/10.1088/1748-9326/abc046

Cottrell, C. (2022). Avoiding a new era in biopiracy: Including indigenous and local knowledge in nature-based solutions to climate change. *Environmental Science & Policy*, *135*, 162-168.https://doi.org/10.1016/j.envsci.2022.05.003

Cousins, J. J. (2021). Justice in nature-based solutions: research and pathways. *Ecological Economics*, 180, 106874. https://doi.org/10.1016/j.ecolecon.2020.106874

Da Veiga Lima, F. A., & de Souza, D. C. (2022). Climate change, seaports, and coastal management in Brazil: an overview of the policy framework. *Regional Studies in Marine Science*, 52, 102365. https://doi.org/10.1016/j.rsma.2022.102365

Dahl, M., McMahon, K., Lavery, P. S., Hamilton, S. H., Lovelock, C. E., & Serrano, O. (2023). Ranking the risk of CO2 emissions from seagrass soil carbon stocks under global change threats. *Global Environmental Change*, 78, 102632. https://doi.org/10.1016/j.gloenvcha.2022.102632

Davies, C., Chen, W. Y., Sanesi, G., & Lafortezza, R. (2021). The European Union roadmap for implementing nature-based solutions: a review. *Environmental Science and Policy*, 121, 49–67. https://doi.org/10.1016/j.envsci.2021.03.018

Davis, J., Guertin, F., Guidry, T., Rogers, M., Saunders, Z., & Uhl, M. (2022). Lessons learned from a corporate manufacturer on driving the adoption of nature-based solutions. *Integrated Environmental Assessment and Management*, 18(1), 74–81. https://doi.org/10.1002/ieam.4442

De Noia I., Favargiotti S., & Marzadri A. (2022). Renaturalising lands as an adaptation strategy. Towards an integrated water-based design approach. *TeMA - Journal of Land Use, Mobility and Environment*, 15(2), 263-286. https://doi.org/10.6093/1970-9870/9074

Debele, S. E., Kumar, P., Sahani, J., Marti-Cardona, B., Mickovski, S. B., Leo, L. S., Porcù, F., Bertini, F., Montesi, D., Vojinovic, Z., & Di Sabatino, S. (2019). Nature-based solutions for hydro-meteorological hazards: revised concepts, classification schemes and databases. *Environmental Research*, 179, 108799. https://doi.org/10.1016/j.envres.2019.108799

Deely, J., Hynes, S., Barquín, J., Burgess, D., Finney, G., Silió, A., Álvarez-Martínez, J. M., Bailly, D., & Ballé-Béganton, J. (2020). Barrier identification framework for the implementation of blue and green infrastructures. *Land Use Policy*, 99., 105108 https://doi.org/10.1016/j.landusepol.2020.105108

Derkzen, M. L., van Teeffelen, A. J. A., & Verburg, P. H. (2017). Green infrastructure for urban climate adaptation: how do residents' views on climate impacts and green infrastructure shape adaptation preferences? *Landscape and Urban Planning*, 157, 106–130. https://doi.org/10.1016/j.landurbplan.2016.05.027

Destoumieux-Garzón, D., Matthies-Wiesler, F., Bierne, N., Binot, A., Boissier, J., Devouge, A., Garric, J., Gruetzmacher, K., Grunau, C., Guégan, J.-F., Hurtrez-Boussès, S., Huss, A., Morand, S., Palmer, C., Sarigiannis, D., Vermeulen, R., & Barouki, R. (2022). Getting out of crises: environmental, social-ecological and evolutionary research is needed to avoid future risks of pandemics. *Environment International*, 158, 106915. https://doi.org/10.1016/j.envint.2021.106915

Ding, Y. J., Li, C. Y., Xiaoming, W. A. N. G., Wang, Y., Wang, S. X., Chang, Y. P., ... & Wang, Z. R. (2021). An overview of climate change impacts on the society in China. *Advances in Climate Change Research*, 12(2), 210-223. https://doi.org/10.1016/j.accre.2021.03.002

Doelle, M., & Puthucherril, T. G. (2023). Nature-based solutions to sea level rise and other climate change impacts on oceanic and coastal environments: a law and policy perspective. *Nordic Journal of Botany*, 2023(1), e03051. https://doi.org/10.1111/njb.03051

Dubo, T., Palomo, I., Camacho, L. L., Locatelli, B., Cugniet, A., Racinais, N., & Lavorel, S. (2023). Nature-based solutions for climate change adaptation are not located where they are most needed across the Alps. *Regional Environmental Change*, 23(1),12. https://doi.org/10.1007/s10113-022-01998-w

Duffaut, C., Versini, P.-A., & Frascaria-Lacoste, N. (2022). Are really nature-based solutions sustainable solutions to design future cities in a context of global change? discussion about the vulnerability of these new solutions and their probable unsustainable implementation. *Science of the Total Environment*, 853, 158535. https://doi.org/10.1016/j.scitotenv.2022.158535

Eekhout, J. P. C., Boix-Fayos, C., Pérez-Cutillas Pedro, & de Vente, J. (2020). The impact of reservoir construction and changes in land use and climate on ecosystem services in a large mediterranean catchment. *Journal of Hydrology*, 590, 125208. https://doi.org/10.1016/j.jhydrol.2020.125208

Eggermont, H., Balian, E., Azevedo José Manuel N, Beumer, V., Brodin, T., Claudet, J., Fady, B., Grube, M., Keune, H., Lamarque, P., Reuter, K., Smith, M., van Ham, C., Weisser, W. W., & Le Roux, X. (2015). Nature-based solutions: new influence for environmental management and research in Europe. *Gaia - Ecological Perspectives for Science and Society*, 24(4), 243–248. https://doi.org/10.14512/gaia.24.4.9

Enu, K. B., Zingraff-Hamed, A., Rahman, M. A., Stringer, L. C., & Pauleit, S. (2023). Potential of nature-based solutions to mitigate hydro-meteorological risks in sub-Saharan Africa. *Natural Hazards and Earth System Sciences*, 23(2), 481-505. https://doi.org/10.5194/nhess-23-481-2023

Epelde, L., Mendizabal, M., Gutiérrez, L., Artetxe, A., Garbisu, C., & Feliu, E. (2022). Quantification of the environmental effectiveness of nature-based solutions for increasing the resilience of cities under climate change. *Urban Forestry & Urban Greening*, 67, 127433. https://doi.org/10.1016/j.ufug.2021.127433

Esperon-Rodríguez, M., Rymer, P. D., Power, S. A., Barton, D. N., Cariñanos, P., Dobbs, C., ... & Tjoelker, M. G. (2022). Assessing climate risk to support urban forests in a changing climate. *Plants, People, Planet,* 4(3), 201-213.https://doi.org/10.1002/ppp3.10240

Faivre, N., Fritz, M., Freitas, T., De Boissezon, B., & Vandewoestijne, S. (2017). Nature-Based Solutions in the EU: Innovating with nature to address social, economic and environmental challenges. *Environmental Research*, *159*, 509-518. https://doi.org/10.1016/j.envres.2017.08.032

Faivre, N., Sgobbi, A., Happaerts, S., Raynal, J., & Schmidt, L. (2018). Translating the Sendai framework into action: the EU approach to ecosystem-based disaster risk reduction. *International Journal of Disaster Risk Reduction*, 32, 4–10. https://doi.org/10.1016/j.ijdrr.2017.12.015

Ferreira, V., Barreira, A. P., Loures, L., Antunes, D., & Panagopoulos, T. (2021). Stakeholders' perceptions of appropriate nature-based solutions in the urban context. *Journal of Environmental Management,* 298, 113502. https://doi.org/10.1016/j.jenvman.2021.113502

Ferreira, V., Barreira, A. P., Pinto, P., & Panagopoulos, T. (2022). Understanding attitudes towards the adoption of nature-based solutions and policy priorities shaped by stakeholders' awareness of climate change. *Environmental Science and Policy*, 131, 149–159. https://doi.org/10.1016/j.envsci.2022.02.007

Field, R. H., Buchanan, G. M., Hughes, A., Smith, P., & Bradbury, R. B. (2020). The value of habitats of conservation importance to climate change mitigation in the UK. *Biological Conservation*, 248, 108619. https://doi.org/10.1016/j.biocon.2020.108619

Fitobór, K., Ulańczyk, R., Kołecka, K., Ramm, K., Włodarek, I., Zima, P., Kalinowska, D., Wielgat, P., Mikulska, M., Antończyk, D., Krzaczkowski, K., Łyszyk, R., & Gajewska, M. (2022). Extreme weather layer method for implementation of nature-based solutions for climate adaptation: case study Słupsk. *Science of the Total Environment*, 842, 156741. https://doi.org/10.1016/j.scitotenv.2022.156751

Fougères, D., Jones, M., McElwee, P. D., Andrade, A., & Edwards, S. R. (2022). Transformative conservation of ecosystems. *Global Sustainability*, 5, e5. https://doi.org/10.1017/sus.2022.4

Frantzeskaki, N. (2019). Seven lessons for planning nature-based solutions in cities. *Environmental Science and Policy*, 93, 101–111. https://doi.org/10.1016/j.envsci.2018.12.033

Frantzeskaki, N., McPhearson, T., Collier, M. J., Kendal, D., Bulkeley, H., Dumitru, A., ... & Pintér, L. (2019). Nature-based solutions for urban climate change adaptation: linking science, policy, and practice communities for evidence-based decision-making. *BioScience*, *69*(6), 455-466. https://doi.org/10.1093/biosci/biz042

Frantzeskaki, N., Vandergert, P., Connop, S., Schipper, K., Zwierzchowska, I., Collier, M., & Lodder, M. (2020). Examining the policy needs for implementing nature-based solutions in cities: findings from city-wide transdisciplinary experiences in Glasgow (UK), Genk (Belgium) and Poznan (Poland). *Land Use Policy*, 96, 104688. https://doi.org/10.1016/j.landusepol.2020.104688

Frantzeskaki, N., & Bush, J. (2021). Governance of nature-based solutions through intermediaries for urban transitions - a case study from Melbourne, Australia. *Urban Forestry & Urban Greening*, 64, 127262. https://doi.org/10.1016/j.ufug.2021.127262

Frantzeskaki, N., Oke, C., Barnett, G., Bekessy, S., Bush, J., Fitzsimons, J., Ignatieva, M., Kendal, D., Kingsley, J., Mumaw, L., & Ossola, A. (2022). A transformative mission for prioritising nature in Australian cities. *Ambio*, 51(6), 1433–1445. https://doi.org/10.1007/s13280-022-01725-z

Gain, A. K., Rahman, M. M., Sadik, M. S., Adnan, M. S. G., Ahmad, S., Ahsan, S. M. M., Ashik-Ur-Rahman, M., Balke, T., Datta, D. K., Dewan, C., Huq, N., Khan, M. S. A., Large, A., Mallick, B., Mohibbullah, M., Mondal, M. S., Narayan, S., Rabbani, G., Rahman, R., … van Loon-Steensma, J. M. (2022). Overcoming challenges for implementing nature-based solutions in deltaic environments: insights from the Ganges-Brahmaputra delta in Bangladesh. *Environmental Research Letters*, 17(6). 064052. https://doi.org/10.1088/1748-9326/ac740a

Galli, A., Peruzzi, C., Beltrame, L., Cislaghi, A., & Masseroni, D. (2021). Evaluating the infiltration capacity of degraded vs. rehabilitated urban greenspaces: lessons learnt from a real-world Italian case study. *Science of the Total Environment*, 787, 147612. https://doi.org/10.1016/j.scitotenv.2021.147612

García Sánchez, F., & Govindarajulu, D. (2023). Integrating blue-green infrastructure in urban planning for climate adaptation: lessons from Chennai and Kochi, India. *Land Use Policy*, 124, 106455. https://doi.org/10.1016/j.landusepol.2022.106455

Glenk, K., Faccioli, M., Martin-Ortega, J., Schulze, C., & Potts, J. (2021). The opportunity cost of delaying climate action: peatland restoration and resilience to climate change. *Global Environmental Change*, 70, 102323. https://doi.org/10.1016/j.gloenvcha.2021.102323

Gómez Martín, E., Máñez Costa, M., & Schwerdtner Máñez, K. (2020a). An operationalized classification of nature-based solutions for water-related hazards: from theory to practice. *Ecological Economics*, 167, 106460. https://doi.org/10.1016/j.ecolecon.2019.106460.

Gómez Martín, E., Giordano, R., Pagano, A., van der Keur, P., & Máñez Costa, M. (2020b). Using a system thinking approach to assess the contribution of nature-based solutions to sustainable development goals. *Science of the Total Environment*, 738, 139693. https://doi.org/10.1016/j.scitotenv.2020.139693

Gómez Martín, E., Máñez Costa, M., Egerer, S., & Schneider, U. A. (2021). Assessing the long-term effectiveness of nature-based solutions under different climate change scenarios. *Science of the Total Environment*, 794, 148515. https://doi.org/10.1016/j.scitotenv.2021.148515

Goodwin, S., Olazabal, M., Castro, A.J., Pascual, U. (2023). Global mapping of urban nature-based solutions for climate change adaptation. *Nature Sustainability* 6(4), 458–469. https://doi.org/10.1038/s41893-022-01036-x.

Gordon, J. E., Crofts, R., Díaz-Martínez Enrique, & Woo, K. S. (2018). Enhancing the role of geoconservation in protected area management and nature conservation. *Geoheritage*, 10(2), 191–203. https://doi.org/10.1007/s12371-017-0240-5

Gordon, J. E., Wignall, R. M. L., Brazier, V., Crofts, R., & Tormey, D. (2022). Planning for climate change impacts on geoheritage interests in protected and conserved areas. *Geoheritage*, *14*(4). https://doi.org/10.1007/s12371-022-00753-1

Griscom, B. W., Adams, J., Ellis, P. W., Houghton, R. A., Lomax, G., Miteva, D. A., Schlesinger, W. H., Shoch, D., Siikamäki, J. V., Smith, P., Woodbury, P., Zganjar, C., Blackman, A., Campari, J., Conant, R. T., Delgado, C., Elias, P., Gopalakrishna, T., Hamsik, M. R., … Fargione, J. (2017). Natural climate solutions. *Proceedings of the National Academy of Sciences*, 114(44), 11645–11650. https://doi.org/10.1073/pnas.1710465114

Groß, E., Mård Johanna, Kalantari, Z., & Bring, A. (2018). Links between Nordic and arctic hydroclimate and vegetation changes: contribution to possible landscape-scale nature-based solutions. *Land Degradation & Development*, 29(10), 3663–3673. https://doi.org/10.1002/ldr.3115

Haase, D., Kabisch, S., Haase, A., Andersson, E., Banzhaf, E., Baró, F., Brenck, M., Fischer, L. K., Frantzeskaki, N., Kabisch, N., Krellenberg, K., Kremer, P., Kronenberg, J., Larondelle, N., Mathey, J., Pauleit, S., Ring, I., Rink, D., Schwarz, N., & Wolff, M. (2017). Greening cities - to

be socially inclusive? About the alleged paradox of society and ecology in cities. *Habitat International*, 64, 41–48. https://doi.org/10.1016/j.habitatint.2017.04.005

Haase, D., Larondelle, N., Andersson, E., Artmann, M., Borgström, S., Breuste, J., et al. (2014). A Quantitative Review of Urban Ecosystem Service Assessments: Concepts, Models, and Implementation. *Ambio*, 43, 413–433 (2014). https://doi.org/10.1007/s13280-014-0504-0.

Haase, A., Koprowska, K., & Borgström, S. (2022). Green regeneration for more justice? an analysis of the purpose, implementation, and impacts of greening policies from a justice perspective in Lódź stare polesie (Poland) and Leipzig's inner east (Germany). *Environmental Science and Policy*, 136, 726–737. https://doi.org/10.1016/j.envsci.2022.08.001

Han, W., & Chen, W. Y. (2022). Embedding nature-based solutions into the social cost of carbon. *Environment International*, 167, 107431.https://doi.org/10.1016/j.envint.2022.107431

Hemati, H., & Abrishamchi, A. (2021). Water allocation using game theory under climate change impact (case study: Zarinehrood). *Journal of Water and Climate Change*, 12(3), 759-771. https://doi.org/10.2166/wcc.2020.153

Hernández-Morcillo, M., Burgess, P., Mirck, J., Pantera, A., & Plieninger, T. (2018). Scanning agroforestry-based solutions for climate change mitigation and adaptation in Europe. *Environmental Science & Policy*, 80, 44-52. https://doi.org/10.1016/j.envsci.2017.11.013

Hobbie, S. E., & Grimm, N. B. (2020). Nature-based approaches to managing climate change impacts in cities. *Philosophical Transactions of the Royal Society B*, 375(1794), 20190124. https://doi.org/10.1098/rstb.2019.0124

Holden, P. B., Rebelo, A. J., Wolski, P., Odoulami, R. C., Lawal, K. A., Kimutai, J., Nkemelang, T., & New, M. G. (2022). Nature-based solutions in mountain catchments reduce impact of anthropogenic climate change on drought streamflow. *Communications Earth & Environment*, 3(1), 51. https://doi.org/10.1038/s43247-022-00379-9

Hoyle, H. E. (2021). Climate-adapted, traditional or cottage-garden planting? Public perceptions, values and socio-cultural drivers in a designed garden setting. *Urban Forestry & Urban Greening*, 65, 127362. https://doi.org/10.1016/j.ufug.2021.127362

Hoyos-Santillan, J., Miranda, A., Lara, A., Sepulveda-Jauregui, A., Zamorano-Elgueta, C., Gómez-González, S., Vásquez-Lavín, F., Garreaud, R. D., & Rojas, M. (2021). Diversifying Chile's climate action away from industrial plantations. *Environmental Science & Policy*, 124, 85–89. https://doi.org/10.1016/j.envsci.2021.06.013

Huang, Y., Tian, Z., Ke, Q., Liu, J., Irannezhad, M., Fan, D., Hou, M., & Sun, L. (2020). Nature-based solutions for urban pluvial flood risk management. *Wiley Interdisciplinary Reviews: Water*, 7(3), e1421. https://doi.org/10.1002/wat2.1421

Jin, L., Yi, Y., & Xu, J. (2020). Forest carbon sequestration and China's potential: the rise of a nature-based solution for climate change mitigation. *China Economic Journal*, 13(2), 200-222. https://doi.org/10.1080/17538963.2020.1754606

Jordan, P., Döring, M., Fröhle, P., & Ratter, B. M. (2023). Exploring past and present dynamics of coastal protection as possible signposts for the future? A case study on the Islands of Amrum and Föhr in the North Frisian Wadden Sea (GER). *Journal of Coastal Conservation*, 27(1), 2. https://doi.org/10.1007/s11852-022-00921-z

Kabisch, N., Frantzeskaki, N., Pauleit, S., Naumann, S., Davis, M., Artmann, M., Haase, D.,... & Bonn, A. (2016). Nature-based solutions to climate change mitigation and adaptation in urban areas: perspectives on indicators, knowledge gaps, barriers, and opportunities for action. *Ecology and Society*, *21*(2). https://doi.org/10.5751/ES-08373-210239

Kabisch, N., van den Bosch, M., & Lafortezza, R. (2017). The health benefits of nature-based solutions to urbanization challenges for children and the elderly–A systematic review. *Environmental Research*, 159, 362-373.https://doi.org/10.1016/j.envres.2017.08.004

Kabisch, N., Frantzeskaki, N., & Hansen, R. (2022). Principles for urban nature-based solutions. *Ambio*, *51*(6), 1388–1401.https://doi.org/10.1007/s13280-021-01685-w

Kattel, G. R., & Wu, C. (2023). Reconfiguration of ecohydrology as a sustainability tool for Himalayan waterways. *Ecohydrology*, 17(4), e2522. https://doi.org/10.1002/eco.2522

Keesstra, S., Nunes, J., Novara, A., Finger, D., Avelar, D., Kalantari, Z., & Cerdà, A. (2018). The superior effect of nature-based solutions in land management for enhancing ecosystem services. *Science of the Total Environment*, 610-611, 997–1009. https://doi.org/10.1016/j.scitotenv.2017.08.077

Keith, H., Vardon, M., Obst, C., Young, V., Houghton, R. A., & Mackey, B. (2021). Evaluating nature-based solutions for climate mitigation and conservation requires comprehensive carbon accounting. *Science of the Total Environment*, 769, 144341. https://doi.org/10.1016/j.scitotenv.2020.144341

Kiedrzyńska, E., Belka, K., Jarosiewicz, P., Kiedrzyński, M., & Zalewski, M. (2021). The enhancement of valley water retentiveness in climate change conditions. *Science of the Total Environment*, 799, 149427. https://doi.org/10.1016/j.scitotenv.2021.149427

Kiss, B., Sekulova, F., Hörschelmann, K., Salk, C. F., Takahashi, W., & Wamsler, C. (2022). Citizen participation in the governance of nature-based solutions. *Environmental Policy and Governance*, *32*(3), 247-272. https://doi.org/10.1002/eet.1987

Koch, K., Ysebaert, T., Denys, S., & Samson, R. (2020). Urban heat stress mitigation potential of green walls: a review. *Urban Forestry & Urban Greening*, 55, 126843. https://doi.org/10.1016/j.ufug.2020.126843

Kotsila, P., Anguelovski, I., Baró, F., Langemeyer, J., Sekulova, F., & JT Connolly, J. (2021). Nature-based solutions as discursive tools and contested practices in urban nature's neoliberalisation processes. *Environment and Planning E: Nature and Space*, 4(2), 252–274. https://doi.org/10.1177/2514848620901437

Kremer, P., Hamstead, Z., Haase, D., McPhearson, T., Frantzeskaki, N., Andersson, E., Kabisch, N., Larondelle, N., Rall, E. L., Voigt, A., Baró, F., Bertram, C., Gómez-Baggethun, E., Hansen, R., Kaczorowska, A., Kain, J.-H., Kronenberg, J., Langemeyer, J., Pauleit, S., … Elmqvist, T. (2016). Key insights for the future of urban ecosystem services research. *Ecology and Society*, 21(2). https://doi.org/10.5751/ES-08445-210229

Kumar, P., Debele, S. E., Sahani, J., Aragão, L., Barisani, F., Basu, B., Bucchignani, E., Charizopoulos, N., Di Sabatino, S., Domeneghetti, A., Edo, A. S., Finér, L., Gallotti, G., Juch, S., Leo, L. S., Loupis, M., Mickovski, S. B., Panga, D., Pavlova, I., … Zieher, T. (2020). Towards an operationalisation of nature-based solutions for natural hazards. *Science of the Total Environment*, 731, 138855. https://doi.org/10.1016/j.scitotenv.2020.138855

Kvamsås, H. (2022). Co-benefits and conflicts in alternative stormwater planning: blue versus green infrastructure? *Environmental Policy and Governance*, 33(3), 232–244. https://doi.org/10.1002/eet.2017

Lafortezza, R., & Chen, J. (2016). The provision of ecosystem services in response to global change: evidences and applications. *Environmental Research*, 147, 576–579. https://doi.org/10.1016/j.envres.2016.02.018

Lampinen, J., García-Antúnez, O., Olafsson, A. S., Kavanagh, K. C., Gulsrud, N. M., & Raymond, C. M. (2022). Envisioning carbon-smart and just urban green infrastructure. *Urban Forestry & Urban Greening*, 75, 127682. https://doi.org/10.1016/j.ufug.2022.127682

Langergraber, G.; Pucher, B.; Simperler, L.; Kisser, J.; Katsou, E.; Buehler, D. Mateo, M. C. Garcia; Atanasova, N. (2020). Implementing nature-based solutions for creating a resourceful circular city. *Blue-Green Systems*, 2(1), 173–185. https://doi.org/10.2166/bgs.2020.933

Lavorel, S., Colloff, M. J., Locatelli, B., Gorddard, R., Prober, S. M., Gabillet, M., Devaux, C., Laforgue, D., & Peyrache-Gadeau, V. (2019). Mustering the power of ecosystems for adaptation to climate change. *Environmental Science and Policy*, 92, 87–97. https://doi.org/10.1016/j.envsci.2018.11.010

Lin, Z., & Qi, J. (2017). Hydro-dam–A nature-based solution or an ecological problem: The fate of the Tonlé Sap Lake. *Environmental Research*, 158, 24-32. https://doi.org/10.1016/j.envres.2017.05.016

Liquete, C., Udias, A., Conte, G., Grizzetti, B., & Masi, F. (2016). Integrated valuation of a nature-based solution for water pollution control. highlighting hidden benefits. E*cosystem Services: Part B*, 22, 392–401. https://doi.org/10.1016/j.ecoser.2016.09.011

Liski, A. H., Ambros, P., Metzger, M. J., Nicholas, K. A., Wilson, A. M. W., & Krause, T. (2019). Governance and stakeholder perspectives of managed re-alignment: adapting to sea level rise in the inner forth estuary, Scotland. *Regional Environmental Change*, 19(8), 2231–2243. https://doi.org/10.1007/s10113-019-01505-8

Liu, L. (2017). Climate resilience strategies of Beijing and Copenhagen and their links to sustainability. *Water Policy*, 19(6), 997–1013. https://doi.org/10.2166/wp.2017.165

Liu, H., Kong, F., Yin, H., Middel, A., Zheng, X., Huang, J., Xu, H., Wang, D., & Wen, Z. (2021). Impacts of green roofs on water, temperature, and air quality: a bibliometric review. *Building and Environment*, 196, 107794. https://doi.org/10.1016/j.buildenv.2021.107794

Ma, S., Wang, H.-Y., Zhang, X., Wang, L.-J., & Jiang, J. (2022). A nature-based solution in forest management to improve ecosystem services and mitigate their trade-offs. *Journal of Cleaner Production*, 351, 131557. https://doi.org/10.1016/j.jclepro.2022.131557

Mabon, L., & Shih, W.-Y. (2021). Urban greenspace as a climate change adaptation strategy for subtropical Asian cities: a comparative study across cities in three countries. *Global Environmental Change*, 68. https://doi.org/10.1016/j.gloenvcha.2021.102248

MacKinnon, K., Richardson, K., & MacKinnon, J. (2020). Protected and other conserved areas: ensuring the future of forest biodiversity in a changing climate. *International Forestry Review*, 22(1), 93-103. https://doi.org/10.1505/146554820829523943

Maes, J., & Jacobs, S. (2017). Nature-based solutions for Europe's sustainable development. *Conservation Letters*, 10(1), 121-124. https://doi.org/10.1111/conl.12216

Maes, M. J. A., Jones, K. E., Toledano, M. B., & Milligan, B. (2019). Mapping synergies and trade-offs between urban ecosystems and the sustainable development goals. *Environmental Science and Policy*, 93, 181–188. https://doi.org/10.1016/j.envsci.2018.12.010

Mahmood, R., Zhang, L., Li, G., & Rahman, M. K. (2021). Geo-based model of intrinsic resilience to climate change: an approach to nature-based solution. *Environment, Development and Sustainability: A Multidisciplinary Approach to the Theory and Practice of Sustainable Development*, 24(10), 11969–11990. https://doi.org/10.1007/s10668-021-01925-9

Mahmood, R., Zhang, L. & Li, G. Assessing effectiveness of nature-based solution with big earth data: 60 years mangrove plantation program in Bangladesh coast. *Ecological Processes* 12(1), 11 (2023). https://doi.org/10.1186/s13717-023-00419-y

Malhi, Y., Franklin, J., Seddon, N., Solan, M., Turner, M. G., Field, C. B., & Knowlton, N. (2020). Climate change and ecosystems: Threats, opportunities and solutions. *Philosophical Transactions of the Royal Society B*, 375(1794), 20190104. https://doi.org/10.1098/rstb.2019.0104

Manes, S., Vale, M. M., Malecha, A., & Pires, A. P. F. (2022). Nature-based solutions promote climate change adaptation safeguarding ecosystem services. *Ecosystem Services*, 55, 101439. https://doi.org/10.1016/j.ecoser.2022.101439

Manes, S., Gama-Maia, D., Vaz, S., Pires, A. P. F., Tardin, R. H., Maricato, G., Bezerra, D. da S., & Vale, M. M. (2023). Nature as a solution for shoreline protection against coastal risks associated with ongoing sea-level rise. *Ocean and Coastal Management*, 235, 106487. https://doi.org/10.1016/j.ocecoaman.2023.106487

Marando, F., Heris, M. P., Zulian, G., Udías, A., Mentaschi, L., Chrysoulakis, N., Parastatidis, D., & Maes, J. (2022). Urban heat island mitigation by green infrastructure in European functional urban areas. *Sustainable Cities and Society*, 77, 103564. https://doi.org/10.1016/j.scs.2021.103564

Markovchick, L. M., Sharma, J., Querejeta, J. I., Swaty, R., Uhey, D. A., Kovacs, Z. I., Johnson, N. C., Whitham, T. G., & Gehring, C. A. (2023). The gap between mycorrhizal science and application: existence, origins, and relevance during the united nation's decade on ecosystem restoration. *Restoration Ecology*, 31(4). https://doi.org/10.1111/rec.13866

Martin, M., Sendra, O., Bastos, A., Bauer, N., Bertram, C., Blenckner, T., . . . Woodcock, J. (2021). Ten new insights in climate science 2021: A horizon scan. *Global Sustainability*, 4, e25. https://doi.org/10.1017/sus.2021.25

Matsler, A. M., Meerow, S., Mell, I. C., & Pavao-Zuckerman, M. A. (2021). A 'green' chameleon: exploring the many disciplinary definitions, goals, and forms of "green infrastructure." *Landscape and Urban Planning*, 214, 104145. https://doi.org/10.1016/j.landurbplan.2021.104145

McPhearson, T., Cook, E. M., Berbés-Blázquez, M., Cheng, C., Grimm, N. B., Andersson, E., Barbosa, O., Chandler, D. G., Chang, H., Chester, M. V., Childers, D. L., Elser, S. R., Frantzeskaki, N., Grabowski, Z., Groffman, P., Hale, R. L., Iwaniec, D. M., Kabisch, N., Kennedy, C., … Troxler, T. G. (2022). A social-ecological-technological systems framework for urban ecosystem services. *One Earth*, 5(5), 505–518. https://doi.org/10.1016/j.oneear.2022.04.007oake

Mehryar, S., & Surminski, S. (2021). National laws for enhancing flood resilience in the context of climate change: potential and shortcomings. *Climate Policy*, 21(2), 133–151. https://doi.org/10.1080/14693062.2020.1808439

Melanidis, M. S., & Hagerman, S. (2022). Competing narratives of nature-based solutions: Leveraging the power of nature or dangerous distraction? *Environmental Science & Policy*, *132*, 273-281. https://doi.org/10.1016/j.envsci.2022.02.028

Meyerhoff, J., Angeli, D., & Hartje, V. (2012). Valuing the benefits of implementing a national strategy on biological diversity—the case of Germany. *Environmental Science and Policy*, 23, 109–119. https://doi.org/10.1016/j.envsci.2012.07.020

Moges, D. M., Kmoch, A., Bhat, H. G., & Uuemaa, E. (2020). Future soil loss in highland Ethiopia under changing climate and land use. *Regional Environmental Change*, 20, 1-14. https://doi.org/10.1007/s10113-020-01617-6

Monteiro, R., & Ferreira, J. C. (2020). Green infrastructure planning as a climate change and risk adaptation tool in coastal urban areas. *Journal of Coastal Research*, 95(SI), 889-893. https://doi.org./10.2112/SI95-173.1

Mori, A. S. (2020). Advancing nature-based approaches to address the biodiversity and climate emergency. *Ecology Letters*, 23(12), 1729–1732. https://doi.org/10.1111/ele.13594

Muyambo, F., Belle, J., Nyam, Y. S., & Orimoloye, I. R. (2022). Climate-change-induced weather events and implications for urban water resource management in the free state province of south Africa. *Environnemental Management*, 71(1), 40–54. https://doi.org/10.1007/s00267-022-01726-4

Næss, J. S., Hu, X., Gvein, M. H., Iordan, C.-M., Cavalett, O., Dorber, M., Giroux, B., & Cherubini, F. (2023). Climate change mitigation potentials of biofuels produced from perennial crops and natural regrowth on abandoned and degraded cropland in Nordic countries. *Journal of Environmental Management*, *325*(Pt A), 116474–116474. https://doi.org/10.1016/j.jenvman.2022.116474

Nassary, E. K., Msomba, B. H., Masele, W. E., Ndaki, P. M., & Kahangwa, C. A. (2022). Exploring urban green packages as part of nature-based solutions for climate change adaptation measures in rapidly growing cities of the global south. *Journal of Environmental Management*, 310, 114786. https://doi.org/10.1016/j.jenvman.2022.114786

Nastran, M., Kobal, M., & Eler, K. (2019). Urban heat islands in relation to green land use in European cities. *Urban Forestry & Urban Greening*, 37, 33–41.https://doi.org/10.1016/j.ufug.2018.01.008

Nesshöver, C., Assmuth, T., Irvine, K. N., Rusch, G. M., Waylen, K. A., Delbaere, B., Haase, D., Jones-Walters, L., Keune, H., Kovacs, E., Krauze, K., Külvik, M., Rey, F., van Dijk, J., Vistad, O. I., Wilkinson, M. E., & Wittmer, H. (2017). The science, policy and practice of nature-based solutions: an interdisciplinary perspective. *The Science of the Total Environment*, *579*, 1215–1227. https://doi.org/10.1016/j.scitotenv.2016.11.106

Niemeyer, J., & Vale, M. M. (2022). Obstacles and opportunities for implementing a policy-mix for ecosystem-based adaptation to climate change in Brazil's Caatinga. *Land Use Policy*, 122, 106385. https://doi.org/10.1016/j.landusepol.2022.106385

Norman, L. M., Lal, R., Wohl, E., Fairfax, E., Gellis, A. C., & Pollock, M. M. (2022). Natural infrastructure in dryland streams (NIDS) can establish regenerative wetland sinks that reverse desertification and strengthen climate resilience. *Science of the Total Environment*, 849,157738. https://doi.org/10.1016/j.scitotenv.2022.157738

Nurhidayah, L., Davies, P., Alam, S., Saintilan, N., & Triyanti, A. (2022). Responding to sea level rise: challenges and opportunities to govern coastal adaptation strategies in Indonesia. *Maritime Studies*, *21*(3), 339–352. https://doi.org/10.1007/s40152-022-00274-1

Oakes, L. E., Cross, M. S., & Zavaleta, E. S. (2021). Rapid assessment to facilitate climate-informed conservation and nature-based solutions. *Conservation Science and Practice*, 3(8), e472. https://doi.org/10.1111/csp2.472

Oakes, L. E., Peterson St-Laurent, G., Cross, M. S., Washington, T., Tully, E., & Hagerman, S. (2022). Strengthening monitoring and evaluation of multiple benefits in conservation initiatives that aim to foster climate change adaptation. *Conservation Science and Practice*, 4(6), e12688. https://doi-org/10.1111/csp2.12688

O'Brien, P., Gunn, J. S., Clark, A., Gleeson, J., Pither, R., & Bowman, J. (2023). Integrating carbon stocks and landscape connectivity for nature-based climate solutions. *Ecology and Evolution*, *13*(1), e9725. https://doi.org/10.1002/ece3.9725

O'Connor, D., Zheng, X., Hou, D., Shen, Z., Li, G., Miao, G., O'Connell, S., & Guo, M. (2019). Phytoremediation: climate change resilience and sustainability assessment at a coastal brownfield redevelopment. *Environment International*, 130, 104945. https://doi.org/10.1016/j.envint.2019.104945

Oral, H. V., Carvalho, P., Gajewska, M., Ursino, N., Masi, F., Hullebusch, E. D. V., ... & Zimmermann, M. (2020). A review of nature-based solutions for urban water management in European circular cities: a critical assessment based on case studies and literature. *Blue-Green Systems*, 2(1), 112-136. https://doi.org/10.2166/bgs.2020.932

Orchard, S., & Schiel, D. R. (2021). Enabling nature-based solutions for climate change on a peri-urban sandspit in Christchurch, New Zealand. *Regional Environmental Change*, 21(3). https://doi.org/10.1007/s10113-021-01791-1

Ordóñez, C., Threlfall, C. G., Kendal, D., Hochuli, D. F., Davern, M., Fuller, R. A., van der Ree, R., & Livesley, S. J. (2019). Urban forest governance and decision-making: a systematic review and synthesis of the perspectives of municipal managers. *Landscape and Urban Planning*, 189, 166–180. https://doi.org/10.1016/j.landurbplan.2019.04.020

Ortiz, A. M. D., de Leon, A. M., Torres, J. N. V., Guiao, C. T. T., & La Viña, A. G. (2021). Implications of COVID-19 on progress in the UN Conventions on Biodiversity and Climate Change. *Global Sustainability*, 4, e11. https://doi.org/doi:10.1017/sus.2021.8

Osaka, S., Bellamy, R., & Castree, N. (2021). Framing "nature-based" solutions to climate change. *Wiley Interdisciplinary Reviews: Climate Change*, 12(5), e729. https://doi.org/10.1002/wcc.729

Ossola, A., & Lin, B. B. (2021). Making nature-based solutions climate-ready for the 50 °c world. *Environmental Science and Policy*, 123, 151–159. https://doi.org/10.1016/j.envsci.2021.05.026

Palomo, I., Locatelli, B., Otero, I., Colloff, M., Crouzat, E., Cuni-Sanchez, A., Gómez-Baggethun, E., González-García, A., Grêt-Regamey, A., Jiménez-Aceituno, A., Martín-López, B., Pascual, U., Zafra-Calvo, N., Bruley, E., Fischborn, M., Metz, R., & Lavorel, S. (2021). Assessing nature-based solutions for transformative change. *One Earth*, 4(5), 730–741. https://doi.org/10.1016/j.oneear.2021.04.013

Pan, H., Page, J., Cong, C., Barthel, S., & Kalantari, Z. (2021). How ecosystems services drive urban growth: Integrating nature-based solutions. *Anthropocene*, 35, 100297. https://doi.org/10.1016/j.ancene.2021.100297

Panno, A., Carrus, G., Lafortezza, R., Mariani, L., & Sanesi, G. (2017). Nature-based solutions to promote human resilience and wellbeing in cities during increasingly hot summers. *Environmental Research*, 159, 249–256. https://doi.org/10.1016/j.envres.2017.08.016

Park, S., Sohn, W., Piao, Y., & Lee, D. (2023). Adaptation strategies for future coastal flooding: performance evaluation of green and grey infrastructure in South Korea. *Journal of Environmental Management*, 334, 117495. https://doi.org/10.1016/j.jenvman.2023.117495

Pasimeni, M. R., Valente, D., Zurlini, G., & Petrosillo, I. (2019). The interplay between urban mitigation and adaptation strategies to face climate change in two European countries. *Environmental Science and Policy*, 95, 20–27. https://doi.org/10.1016/j.envsci.2019.02.002

Peck, A., Adams, S., Armstrong, A., Bartlett, A., Bortman, M., Branco, A., ... & Smith, E. (2022). A new framework for flood adaptation: introducing the Flood Adaptation Hierarchy. *Ecology and Society*, 27(4).https://doi.org/10.5751/ES-13544-270405

Pedersen Zari, M., Kiddle, G. L., Blaschke, P., Gawler, S., & Loubser, D. (2019). Utilising nature-based solutions to increase resilience in Pacific Ocean cities. *Ecosystem Services*, 38, 100968. https://doi.org/10.1016/j.ecoser.2019.100968

Pedersen Zari, M., Blaschke, P. M., Jackson, B., Komugabe-Dixson Aimée, Livesey, C., Loubser, D. I., Martinez-Almoyna Gual, C., Maxwell, D., Rastandeh, A., Renwick, J., Weaver, S., & Archie, K. M. (2020). Devising urban ecosystem-based adaptation projects with developing nations: a case study of Port Vila, Vanuatu. *Ocean and Coastal Management*, 184, 105037. https://doi.org/10.1016/j.ocecoaman.2019.105037

Penny, J., Alves, P. B., De-Silva, Y., Chen, A. S., Djordjević, S., Shrestha, S., & Babel, M. (2023). Analysis of potential nature-based solutions for the Mun River Basin, Thailand. *Water Science and Technology*, 87(6), 1496-1514. https://doi.org/10.2166/wst.2023.050

Pettorelli, N., Graham, N. A. J., Seddon, N., Lowton, M. J., Sutherland, W. J., Koldewey, H. J., Prentice, H. C., & Barlow, J. (2021). Time to integrate global climate change and biodiversity science-policy agendas. *The Journal of Applied Ecology*, 58(11), 2384–2393. https://doi.org/10.1111/1365-2664.13985

Pienaru, A. M., & Rădulescu, D. (2022). Opportunities To Apply Nature-Based Solutions in Romania in the Context Of European Common Agriculture Policy. *Scientific Papers. Series E. Land Reclamation, Earth Observation & Surveying, Environmental Engineering*, 11, 511-516.

Prenner, F., Müller, H., Stern, P., Holzer, M., Rauch, H. P., & Kretschmer, F. (2022). Suitability pre-assessment for decoupling in-sewer captured streams to support urban blue-green climate

adaptation measures. *Journal of Water and Climate Change*, 13(4), 1748-1764. https://doi.org/10.2166/wcc.2022.458

Qi, J. J., & Dauvergne, P. (2022). China and the global politics of nature-based solutions. *Environmental Science and Policy*, *137*, 1–11. https://doi.org/10.1016/j.envsci.2022.08.008

Queirós, A. M., Talbot, E., Beaumont, N. J., Somerfield, P. J., Kay, S., Pascoe, C., Dedman, S., Fernandes, J. A., Jueterbock, A., Miller, P. I., Sailley, S. F., Sará, G., Carr, L. M., Austen, M. C., Widdicombe, S., Rilov, G., Levin, L. A., Hull, S. C., & Walmsley, S. F. (2021). Bright spots as climate-smart marine spatial planning tools for conservation and blue growth. *Global Change Biology*, 27(21), 5514–5531. https://doi.org/10.1111/gcb.15827

Queirós, A. M., Tait, K., Clark, J. R., Bedington, M., Pascoe, C., Torres, R., Somerfield, P. J., & Smale, D. A. (2023). Identifying and protecting macroalgae detritus sinks toward climate change mitigation. *Ecological Applications: A Publication of the Ecological Society of America*, 33(3), 2798. https://doi.org/10.1002/eap.2798

Raw, J. L., Van Niekerk, L., Chauke, O., Mbatha, H., Riddin, T., & Adams, J. B. (2023). Blue carbon sinks in South Africa and the need for restoration to enhance carbon sequestration. *Science of the Total Environment*, 859, 160142. https://doi.org/10.1016/j.scitotenv.2022.160142

Raymond, C. M., Frantzeskaki, N., Kabisch, N., Berry, P., Breil, M., Nita, M. R., Geneletti, D., & Calfapietra, C. (2017). A framework for assessing and implementing the co-benefits of nature-based solutions in urban areas. *Environmental Science and Policy*, *77*, 15–24. https://doi.org/10.1016/j.envsci.2017.07.008

Reed, G., Brunet, N. D., McGregor, D., Scurr, C., Sadik, T., Lavigne, J., & Longboat, S. (2022). Toward indigenous visions of nature-based solutions: an exploration into Canadian federal climate policy. *Climate Policy*, 22(4), 514–533. https://doi.org/10.1080/14693062.2022.2047585

Reu Junqueira, J., Serrao-Neumann, S., & White, I. (2022). Using green infrastructure as a social equity approach to reduce flood risks and address climate change impacts: a comparison of performance between cities and towns. *Cities*, 131, 104051. https://doi.org/10.1016/j.cities.2022.104051

Reu Junqueira, J., Serrao-Neumann, S., & White, I. (2023). Developing and testing a cost-effectiveness analysis to prioritize green infrastructure alternatives for climate change adaptation. *Water and Environment Journal*, 37(2), 242–255. https://doi.org/10.1111/wej.12832

Rifai, H., Quevedo, J. M. D., Lukman, K. M., Sondak, C. F. A., Risandi, J., Hernawan, U. E., Uchiyama, Y., Ambo-Rappe, R., & Kohsaka, R. (2022). Potential of seagrass habitat restorations as nature-based solutions: practical and scientific implications in Indonesia. *Ambio*, 52(3), 546–555. https://doi.org/10.1007/s13280-022-01811-2

Riisager-Simonsen, C., Fabi, G., van Hoof, L., Holmgren, N., Marino, G., & Lisbjerg, D. (2022). Marine nature-based solutions: Where societal challenges and ecosystem requirements meet the potential of our oceans. *Marine Policy*, 144, 105198. https://doi.org/10.1016/j.marpol.2022.105198

Roberts, C. M., O'Leary, B. C., McCauley, D. J., Cury, P. M., Duarte, C. M., Lubchenco, J., Pauly, D., Sáenz-Arroyo, A., Sumaila, U. R., Wilson, R. W., Worm, B., & Castilla, J. C. (2017). Marine

reserves can mitigate and promote adaptation to climate change. *Proceedings of the National Academy of Sciences*, 114(24), 6167–6175. https://doi.org/10.1073/pnas.1701262114

Roberts, C. M., O'Leary, B. C., & Hawkins, J. P. (2020). Climate change mitigation and nature conservation both require higher protected area targets. *Philosophical Transactions of the Royal Society B*, 375(1794), 20190121. https://doi.org/10.1098/rstb.2019.0121

Roberts, M. T., Geris, J., Hallett, P. D., & Wilkinson, M. E. (2023). Mitigating floods and attenuating surface runoff with temporary storage areas in headwaters. *Wiley Interdisciplinary Reviews: Water*, 10(3), e1634. https://doi.org/10.1002/wat2.1634

Rödl, A., & Arlati, A. (2022). A general procedure to identify indicators for evaluation and monitoring of nature-based solution projects. *Ambio,* 51(11), 2278–2293. https://doi.org/10.1007/s13280-022-01740-0

Roe, S., Streck, C., Beach, R., Busch, J., Chapman, M., Daioglou, V., Deppermann, A., Doelman, J., Engelmann, J., Fricko, O., Frischmann, C., Funk, J., Grassi, G., Havlik, P., Hanssen, S., Humpenöder, F., Landholm, D., Lomax, G., Lehmann, J., … Lawrence, D. (2021). Land-based measures to mitigate climate change: potential and feasibility by country. *Global Change Biology*, 27(23), 6025–6058. https://doi.org/10.1111/gcb.15873

Rosenberger, L., Leandro, J., Pauleit, S., & Erlwein, S. (2021). Sustainable stormwater management under the impact of climate change and urban densification. *Journal of Hydrology*, 596, 126137. https://doi.org/10.1016/j.jhydrol.2021.126137

Roy, P. S., Ramachandran, R. M., Paul, O., Thakur, P. K., Ravan, S., Behera, M. D., Sarangi, C., & Kanawade, V. P. (2022). Anthropogenic land use and land cover changes—a review on its environmental consequences and climate change. *Journal of the Indian Society of Remote Sensing*, 50(8), 1615–1640. https://doi.org/10.1007/s12524-022-01569-w

Ruangpan, L., Vojinovic, Z., Di Sabatino, S., Leo, L. S., Capobianco, V., Oen, A. M., ... & Lopez-Gunn, E. (2020). Nature-based solutions for hydro-meteorological risk reduction: a state-of-the-art review of the research area. *Natural Hazards and Earth System Sciences*, *20*(1), 243-270. https://doi.org/10.5194/nhess-20-243-2020

Russo, A., Escobedo, F. J., Cirella, G. T., & Zerbe, S. (2017). Edible green infrastructure: an approach and review of provisioning ecosystem services and disservices in urban environments. *Agriculture, Ecosystems and Environment*, *242*, 53–66. https://doi.org/10.1016/j.agee.2017.03.026

Satori, D., Tovar, C., Faruk, A., Hammond Hunt, E., Muller, G., Cockel, C., ... & Pironon, S. (2022). Prioritising crop wild relatives to enhance agricultural resilience in sub-Saharan Africa under climate change. *Plants, People, Planet*, 4(3), 269-282. https://doi.org/10.1002/ppp3.10247

Scarano, F. R. (2017). Ecosystem-based adaptation to climate change: concept, scalability and a role for conservation science. *Perspectives in Ecology and Conservation*, 15(2), 65-73. https://doi.org/10.1016/j.pecon.2017.05.003

Schneider, P., Pilzecker, C., & Reinstorf, F. (2022). Urban green infrastructure for coping with climate extremes in Holguin: ecological engineering solutions in the Cuban context. *Clean - Soil, Air, Water*, *50*(10), 2000422. https://doi.org/10.1002/clen.202000422

Schulte, I., Eggers, J., Nielsen, J. Ø., & Fuss, S. (2022). What influences the implementation of natural climate solutions? A systematic map and review of the evidence. *Environmental Research Letters*, 17(1), 013002. https://doi.org/10.1088/1748-9326/ac4071

Seddon, N., Turner, B., Berry, P., Chausson, A. and Girandin, C. A. J. (2019). Grounding nature-based climate solutions in sound biodiversity science. *Nature Climate Change* 9(2), 84–87.

Seddon, N., Chausson, A., Berry, P., Girardin, C. A. J., Smith, A., & Turner, B. (2020a). Understanding the value and limits of nature-based solutions to climate change and other global challenges. *Philosophical Transactions of the Royal Society B*, 375(1794), 20190120. https://doi.org/10.1098/rstb.2019.0120

Seddon, N., Daniels, E., Davis, R., Chausson, A., Harris, R., Hou-Jones, X., ... & Wicander, S. (2020b). Global recognition of the importance of nature-based solutions to the impacts of climate change. *Global Sustainability*, 3, e15. https://doi.org/10.1017/sus.2020.8

Seddon, N., Smith, A., Smith, P., Key, I., Chausson, A., Girardin, C., House, J., Srivastava, S., & Turner, B. (2021). Getting the message right on nature-based solutions to climate change. *Global Change Biology*, *27*(8), 1518–1546.https://doi.org/10.1111/gcb.15513

Seddon, N. (2022). Harnessing the potential of nature-based solutions for mitigating and adapting to climate change. *Science*, 376(6600), 1410–1416. https://doi.org/10.1126/science.abn9668

Sekulova, F., Anguelovski, I., Kiss, B., Kotsila, P., Baró, F., Palgan, Y. V., & Connolly, J. (2021). The governance of nature-based solutions in the city at the intersection of justice and equity. *Cities*, *112*, 103136. https://doi.org/10.1016/j.cities.2021.103136

Selomane, O., Reyers, B., Biggs, R., Tallis, H., & Polasky, S. (2015). Towards integrated social-ecological sustainability indicators: exploring the contribution and gaps in existing global data. *Ecological Economics*, 118, 140–146. https://doi.org/10.1016/j.ecolecon.2015.07.024

Sherren, K., Sutton, K., & Chappell, E. (2022). Climax thinking on the coast: a focus group priming experiment with coastal property owners about climate adaptation. *Environmental Management*, 70(3), 475–488. https://doi.org/10.1007/s00267-022-01676-x

Shin, Y. J., Midgley, G. F., Archer, E. R. M., Arneth, A., Barnes, D. K. A., Chan, L., Hashimoto, S., Hoegh-Guldberg, O., Insarov, G., Leadley, P., Levin, L. A., Ngo, H. T., Pandit, R., Pires, A. P. F., Pörtner, H.-O., Rogers, A. D., Scholes, R. J., Settele, J., & Smith, P. (2022). Actions to halt biodiversity loss generally benefit the climate. *Global Change Biology*, 28(9), 2846–2874. https://doi.org/10.1111/gcb.16109

Silva, L. C. R. (2022). Expanding the scope of biogeochemical research to accelerate atmospheric carbon capture. *Biogeochemistry: An International Journal*, *161*(1), 19–40. https://doi.org/10.1007/s10533-022-00957-1

Smith, P., Arneth, A., Barnes, D. K. A., Ichii, K., Marquet, P. A., Popp, A., Rogers, A. D., Scholes, R. J., Strassburg, B., Wu, J., & Ngo, H. (2022). How do we best synergize climate mitigation actions to co-benefit biodiversity? *Global Change Biology*, 28(8), 2555–2577. https://doi.org/10.1111/gcb.16056

Solan, M., Bennett, E. M., Mumby, P. J., Leyland, J., & Godbold, J. A. (2020). Benthic-based contributions to climate change mitigation and adaptation. *Philosophical Transactions of the Royal Society B*, 375(1794), 20190107. https://doi.org/10.1098/rstb.2019.0107

Stankovic, M., Ambo-Rappe, R., Carly, F., Dangan-Galon, F., Fortes, M. D., Hossain, M. S., Kiswara, W., Van Luong, C., Minh-Thu, P., Mishra, A. K., Noiraksar, T., Nurdin, N., Panyawai, J., Rattanachot, E., Rozaimi, M., Soe Htun, U., & Prathep, A. (2021). Quantification of blue carbon in seagrass ecosystems of Southeast Asia and their potential for climate change mitigation. *Science of the Total Environment*, 783, 146858. https://doi.org/10.1016/j.scitotenv.2021.146858

Strauß, L., Baker, T. R., de Lima, R. F., Afionis, S., & Dallimer, M. (2022). Limited integration of biodiversity within climate policy: evidence from the alliance of small island states. *Environmental Science and Policy*, 128, 216–227. https://doi.org/10.1016/j.envsci.2021.11.019

Stroud, H. M., Kirshen, P. H., & Timmons, D. (2022). Monetary evaluation of co-benefits of nature-based flood risk reduction infrastructure to promote climate justice. *Mitigation and Adaptation Strategies for Global Change, 28*(1), 5. https://doi.org/10.1007/s11027-022-10037-2

Su, W., Zhang, L., & Chang, Q. (2022). Nature-based solutions for urban heat mitigation in historical and cultural block: the case of Beijing old city. *Building and Environment*, *225*. https://doi.org/10.1016/j.buildenv.2022.109600

Sun, X., Wang, P., Ferris, T., Lin, H., Dreyfus, G., Gu, B.-H., Zaelke, D., & Wang, Y. (2022). Fast action on short-lived climate pollutants and nature-based solutions to help countries meet carbon neutrality goals. *Advances in Climate Change Research*, *13*(4), 564–577.https://doi.org/10.1016/j.accre.2022.06.003

Sušnik, J., Masia, S., Kravčík, M., Pokorný, J., & Hesslerová, P. (2022). Costs and benefits of landscape-based water retention measures as nature-based solutions to mitigating climate impacts in eastern Germany, Czech Republic, and Slovakia. *Land Degradation & Development*, 33(16), 3074–3087. https://doi.org/10.1002/ldr.4373

Techera, E. J. E. (2023). The intersection of marine and coastal conservation and nature-based solutions to climate change: governance insights from Indian Ocean small island states. *Ocean and Coastal Management*, 239, 106579.https://doi.org/10.1016/j.ocecoaman.2023.106579

Teo, H. C., Zeng, Y., Sarira, T. V., Fung, T. K., Zheng, Q., Song, X. P., ... & Koh, L. P. (2021). Global urban reforestation can be an important natural climate solution. *Environmental Research Letters*, 16(3), 034059. https://doi.org/10.1088/1748-9326/abe783

Thorslund, J., Jarsjo, J., Jaramillo, F., Jawitz, J. W., Manzoni, S., Basu, N. B., Chalov, S. R., Cohen, M. J., Creed, I. F., Goldenberg, R., Hylin, A., Kalantari, Z., Koussis, A. D., Lyon, S. W., Mazi, K., Mard, J., Persson, K., Pietro, J., Prieto, C., … Destouni, G. (2017). Wetlands as large-scale nature-based solutions: status and challenges for research, engineering and management. *Ecological Engineering*, 108, 489–497. https://doi.org/10.1016/j.ecoleng.2017.07.012

Tito, R., Salinas, N., Cosio, E. G., Espinoza, T. E. B., Muñiz, J. G., Aragón, S., ... & Roman-Cuesta, R. M. (2022). Secondary forests in Peru: differential provision of ecosystem services compared to other post-deforestation forest transitions. *Ecology and Society*, 27(3). https://doi.org/10.5751/ES-13446-270312

Toxopeus, H., Kotsila, P., Conde, M., Katona, A., van der Jagt, A. P. N., & Polzin, F. (2020). How 'just' is hybrid governance of urban nature-based solutions? *Cities*, 105, 102839. https://doi.org/10.1016/j.cities.2020.102839

Turkelboom, F., Demeyer, R., Vranken, L., De Becker, P., Raymaekers, F., & De Smet, L. (2021). How does a nature-based solution for flood control compare to a technical solution? Case study evidence from Belgium. *Ambio,* 50(8), 1431–1445. https://doi.org/10.1007/s13280-021-01548-4

Turkelboom, F., M. Leone, S. Jacobs, E. Kelemen, M. García-Llorente, F. Baró, M. Termansen, D. N. Barton, *et al.* (2018). When we cannot have it all : Ecosystem services tradeoffs in the context of spatial planning. *Ecosystem Services* 29: 566–578. https://doi.org/10.1016/j.ecoser.2017.10.011

Turner, W. R. (2018). Looking to nature for solutions. *Nature Climate Change*, 8(1), 18-19. https://doi.org/10.1038/s41558-017-0048-y

Turner, B., Devisscher, T., Chabaneix, N., Woroniecki, S., Messier, C., & Seddon, N. (2022). The role of nature-based solutions in supporting social-ecological resilience for climate change adaptation. *Annual Review of Environment and Resources*, 47, 123-148. https://doi.org/10.1146/annurev-environ-012220-010017

Twohig, C., Casali, Y., & Aydin, N. Y. (2022). Can green roofs help with stormwater floods? A geospatial planning approach. *Urban Forestry & Urban Greening*, 76, 127724. https://doi.org/10.1016/j.ufug.2022.127724

Tyllianakis, E., Martin-Ortega, J., & Banwart, S. A. (2022). An approach to assess the world's potential for disaster risk reduction through nature-based solutions. *Environmental Science & Policy*, 136, 599–608. https://doi.org/10.1016/j.envsci.2022.07.021

Tzoulas, K., Galan, J., Venn, S., Dennis, M., Pedroli, B., Mishra, H., Haase, D., Pauleit, S., Niemelä, J., & James, P. (2021). A conceptual model of the social–ecological system of nature-based solutions in urban environments. *Ambio*, 50(2), 335-345. https://doi.org/10.1007/s13280-020-01380-2

Van den Bosch, M., & Sang, Å. O. (2017). Urban natural environments as nature-based solutions for improved public health–A systematic review of reviews. *Environmental Research*, *158*, 373-384.https://doi.org/ 10.1016/j.envres.2017.05.040

Vasiliev, D., & Greenwood, S. (2022). Making green pledges support biodiversity: nature-based solution design can be informed by landscape ecology principles. *Land Use Policy*, 117, 106129. https://doi.org/10.1016/j.landusepol.2022.106129

Vieira, J., Matos, P., Mexia, T., Centro de Estudos Florestais, I. S. de A. U. de L. E. P. A. G. T. da A. 1349-017 L. (P., Silva, P., Lopes, N., Freitas, C., Correia, O., Santos-Reis, M., Branquinho, C., Pinho, P., & Centro de Recursos Naturais e Ambiente, I. S. T. U. de L. A. R. P. 1049-001 L. (P. (2018). Green spaces are not all the same for the provision of air purification and climate regulation services: the case of urban parks. *Environmental Research*, 160, 306-313. https://doi.org/10.1016/J.ENVRES.2017.10.006

Vignola, R., Martinez, C., Imbach, P., & Locatelli, B. (2009). Ecosystem-based adaptation to climate change: what role for policymakers, society and scientists? *Mitigation and Adaptation Strategies for Global Change*, *14*(8), 691–696. https://doi.org/10.1007/s11027-009-9193-6

Vojinovic, Z., Alves, A., Gómez, J. P., Weesakul, S., Keerakamolchai, W., Meesuk, V., & Sanchez, A. (2021). Effectiveness of small- and large-scale nature-based solutions for flood mitigation: the case of Ayutthaya, Thailand. *Science of the Total Environment*, 789, 147725. https://doi.org/10.1016/j.scitotenv.2021.147725

Von Döhren, P., Haase, D. (2015). Ecosystem disservices research: A review of the state of the art with a focus on cities. *Ecological Indicators*, 52, 490-497. https://doi.org/10.1016/j.ecolind.2014.12.027

Wamsler, C., Niven, L., Beery, T. H., Bramryd, T., Ekelund, N., Jönsson, K. I., ... & Stålhammar, S. (2016). Operationalizing ecosystem-based adaptation: harnessing ecosystem services to buffer communities against climate change. *Ecology and Society*, 21(1). https://doi.org/10.5751/ES-08266-210131

Wamsler, C., & Raggers, S. (2018). Principles for supporting city-citizen commoning for climate adaptation: from adaptation governance to sustainable transformation. *Environmental Science and Policy*, 85, 81–89. https://doi.org/10.1016/j.envsci.2018.03.021

Wamsler, C., Wickenberg, B., Hanson, H., Alkan Olsson, J., Stålhammar, S., Björn, H., Falck, H., Gerell, D., Oskarsson, T., Simonsson, E., Torffvit, F., & Zelmerlow, F. (2020a). Environmental and climate policy integration: targeted strategies for overcoming barriers to nature-based solutions and climate change adaptation. *Journal of Cleaner Production*, 247, 119154. https://doi.org/10.1016/j.jclepro.2019.119154

Wamsler, C., Alkan-Olsson, J., Björn H, Falck, H., Hanson, H., Oskarsson, T., Simonsson, E., & Zelmerlow, F. (2020b). Beyond participation: when citizen engagement leads to undesirable outcomes for nature-based solutions and climate change adaptation. *Climatic Change: An Interdisciplinary, International Journal Devoted to the Description, Causes and Implications of Climatic Change*, 158(2), 235–254. https://doi.org/10.1007/s10584-019-02557-9

Wamsler, C., Osberg, G., Panagiotou, A., Smith, B., Stanbridge, P., Osika, W., & Mundaca, L. (2022). Meaning-making in a context of climate change: supporting agency and political engagement. *Climate Policy*, 23(7), 829–844. https://doi.org/10.1080/14693062.2022.2121254

Wang, B., Waters, C., Anwar, M. R., Cowie, A., Liu, D. L., Summers, D., Paul, K., & Feng, P. (2022). Future climate impacts on forest growth and implications for carbon sequestration through reforestation in southeast Australia. *Journal of Environmental Management*, 302(Pt A), 113964. https://doi.org/10.1016/j.jenvman.2021.113964

Wedding, L. M., Moritsch, M., Verutes, G., Arkema, K., Hartge, E., Reiblich, J., Douglass, J., Taylor, S., & Strong, A. L. (2021). Incorporating blue carbon sequestration benefits into sub-national climate policies. *Global Environmental Change*, 69, 102206. https://doi.org/10.1016/j.gloenvcha.2020.102206

Wellmann, T., Andersson, E., Knapp, S., Lausch, A., Palliwoda, J., Priess, J., Scheuer, S., & Haase, D. (2022). Reinforcing nature-based solutions through tools providing social-ecological-technological integration. *Ambio*, *52*(3), 489–507.https://doi.org/10.1007/s13280-022-01801-4

Wickenberg, B., McCormick, K., & Olsson, J. A. (2021). Advancing the implementation of nature-based solutions in cities: a review of frameworks. *Environmental Science and Policy*, 125, 44–53. https://doi.org/10.1016/j.envsci.2021.08.016

Williams, M., Reay, D., & Smith, P. (2023). Avoiding emissions versus creating sinks-effectiveness and attractiveness to climate finance. *Global Change Biology*, *29*(8), 2046–2049. https://doi.org/10.1111/gcb.16598

Wong, F. W. M. H., Foley, A., Del Rio, D. F., Rooney, D., Shariff, S., Dolfi, A., & Srinivasan, G. (2022). Public perception of transitioning to a low-carbon nation: a Malaysian scenario. *Clean*

Technologies and Environmental Policy: Focusing on Technology Research, Innovation, Demonstration, Insights and Policy Issues for Sustainable Technologies, *24*(10), 3077–3092. https://doi.org/10.1007/s10098-022-02345-7

Woroniecki, S., Wamsler, C., & Boyd, E. (2019). The promises and pitfalls of ecosystem-based adaptation to climate change as a vehicle for social empowerment. *Ecology and Society*, 24(2). https://www.jstor.org/stable/26796957

Woroniecki, S., Wendo, H., Brink, E., Islar, M., Krause, T., Vargas, A.-M., & Mahmoud, Y. (2020). Nature unsettled: how knowledge and power shape 'nature-based' approaches to societal challenges. *Global Environmental Change*, 65, 102132. https://doi.org/10.1016/j.gloenvcha.2020.102132

Woroniecki, S., Spiegelenberg, F. A., Chausson, A., Turner, B., Key, I., Md. Irfanullah, H., & Seddon, N. (2023). Contributions of nature-based solutions to reducing people's vulnerabilities to climate change across the rural Global South. *Climate and Development*, 15(7), 590-607. https://doi.org/10.1080/17565529.2022.2129954

Wüstemann, H., Meyerhoff, J., Rühs, M., Schäfer, A., & Hartje, V. (2014). Financial costs and benefits of a program of measures to implement a national strategy on biological diversity in Germany. *Land Use Policy*, 36, 307–318. https://doi.org/10.1016/j.landusepol.2013.08.009

Xie, L., & Bulkeley, H. (2020). Nature-based solutions for urban biodiversity governance. *Environmental Science and Policy*, 110, 77–87. https://doi.org/10.1016/j.envsci.2020.04.002

Xie, L., Bulkeley, H., & Tozer, L. (2022). Mainstreaming sustainable innovation: unlocking the potential of nature-based solutions for climate change and biodiversity. *Environmental Science and Policy*, 132, 119–130. https://doi.org/10.1016/j.envsci.2022.02.017

Yazar, M., & York, A. (2022). Disentangling justice as recognition through public support for local climate adaptation policies: insights from the southwest us. *Urban Climate*, *41,* 101079. https://doi.org/10.1016/j.uclim.2021.101079

Young, A. F., Marengo, J. A., Martins Coelho, J. O., Scofield, G. B., de Oliveira Silva, C. C., & Prieto, C. C. (2019). The role of nature-based solutions in disaster risk reduction: the decision maker's perspectives on urban resilience in São Paulo state. *International Journal of Disaster Risk Reduction*, 39, 101219. https://doi.org/10.1016/j.ijdrr.2019.101219

Young, M. A., Serrano, O., Macreadie, P. I., Lovelock, C. E., Carnell, P., & Ierodiaconou, D. (2021). National scale predictions of contemporary and future blue carbon storage. *Science of the Total Environment*, 800, 149573. https://doi.org/10.1016/j.scitotenv.2021.149573

Zaimes, G. N. (2020). Mediterranean riparian areas-climate change implications and recommendations. *Journal of Environmental Biology*, 41(5), 957–965. https://doi.org/10.22438/JEB//41/5/MRN-1454

Zhong, C., Li, T., Bi, R., Sanganyado, E., Huang, J., Jiang, S., ... & Du, H. (2023). A systematic overview, trends and global perspectives on blue carbon: A bibliometric study (2003–2021). *Ecological Indicators*, *148*, 110063.https://doi.org/10.1016/j.ecolind.2023.110063

Zingraff-Hamed, A., Hüesker F., Albert, C., Brillinger, M., Huang, J., Lupp, G., Scheuer, S., Schlätel Mareen, & Schröter Barbara. (2021). Governance models for nature-based solutions:

seventeen cases from Germany. *Ambio*, *50*(8), 1610–1627. https://doi.org/10.1007/s13280-020-01412-x

Zölch, T., Henze, L., Keilholz, P., & Pauleit, S. (2017). Regulating urban surface runoff through nature-based solutions - an assessment at the micro-scale. *Environmental Research*, 157, 135–144. https://doi.org/10.1016/j.envres.2017.05.023

Zölch, T., Wamsler, C., & Pauleit, S. (2018). Integrating the ecosystem-based approach into municipal climate adaptation strategies: the case of Germany. *Journal of Cleaner Production*, 170, 966–977. https://doi.org/10.1016/j.jclepro.2017.09.146

Zwierzchowska, I., Fagiewicz, K., Poniży, L., Lupa, P., & Mizgajski, A. (2019). Introducing nature-based solutions into urban policy - facts and gaps. Case study of Poznan. *Land Use Policy*, 85, 161–175. https://doi.org/10.1016/j.landusepol.2019.03.025
